# Floral and pollinator functional diversity mediate network structure along an elevational gradient

**DOI:** 10.1007/s00035-024-00308-w

**Published:** 2024-03-16

**Authors:** Luis A. Aguirre, Robert R. Junker

**Affiliations:** 1 Department of Environment and Biodiversity, University of Salzburg, Salzburg, Austria; 2 Department of Biology, University of Massachusetts, Amherst, MA USA; 3 Graduate Program in Organismic and Evolutionary Biology, University of Massachusetts, Amherst, MA USA; 4 Evolutionary Ecology of Plants, Department of Biology, University of Marburg, Marburg, Germany

**Keywords:** Alpine communities, Bipartite networks, Dynamic range boxes, Eltonian niches, Mutualism

## Abstract

Elevational gradients in alpine ecosystems are well suited to study how plant and pollinator communities respond to climate change. In the Austrian Alps, we tested how the taxonomic and functional diversity of plants and their pollinators change with increasing elevation and how this affects plant–pollinator network structure. We measured the phenotypes of flowering plants and their pollinators and observed their interactions in 24 communities along an elevational gradient. To assess variation in floral and pollinator traits, we then quantified trait spaces (*n*-dimensional hypervolumes) occupied by flowers and pollinators in each community. To assess plant–pollinator network structure, we quantified the levels of complementary specialization (H_2_’), modularity and nestedness (weighted NODF) for each community. As elevation increased, most measures of diversity and network specialization either declined linearly or in an oscillating manner. Communities that exhibited higher pollinator functional diversity exhibited larger degrees of complementary specialization and modularity; and communities with greater floral and pollinator functional diversity and higher phylogenetic diversity were less nested. Altogether, the degree to which elevation, species diversity, functional diversity and network structure changed in conjunction suggests environmental effects on the functional and phylogenetic diversity of plants and pollinators and consequently network structure. Our results suggest that the effects of climate change on plant and pollinator community composition will impact plant–pollinator network structure and potentially pollination services at the community scale.

**Supplementary Information:**

The online version contains supplementary material available at 10.1007/s00035-024-00308-w.

## Introduction

Climate change will have significant consequences for the composition of flowering plant and pollinator communities and likely the patterns in which plants and pollinators interact (Tylianakis and Morris 2017). Elevational gradients in alpine ecosystems are well suited to study the responses of plant and pollinator communities to climate change because they exhibit a wide range of climatic conditions occurring over relatively small spatial scales that can be used as space-for-time proxies (Sundqvist et al. 2013). This variation in climatic conditions manifests in varying levels of plant and pollinator diversity (Inouye 2020), and possibly how plants and pollinators interact. By observing the relationship along elevational gradients between plant and pollinator diversity, and plant–pollinator interaction patterns (i.e., network structure), it may be possible to elucidate underlying mechanisms shaping these interactions (Kaiser-Bunbury et al. 2017; Woodcock et al. 2019).

The relationships between elevation and plant and pollinator diversity are well-defined, but the link between elevation and plant–pollinator network structure remains understudied and is characterized by inconsistent patterns. Plant and pollinator taxonomic diversity either decrease along an elevational gradient in a linear manner or exhibit a unimodal pattern (Hoiss et al. 2012, 2015; Lara-Romero et al. 2019; Minachilis et al. 2023), as does their functional diversity (Pellissier et al. 2010; Junker and Larue-Kontic 2018; Lara-Romero et al. 2019). As plant and pollinator diversity vary along an elevational gradient, it is expected that concurrent changes in network structure would also manifest (Minachilis et al. 2023). Plant–pollinator networks at higher elevations have been shown to be less specialized and less modular (Hoiss et al. 2015; Lara-Romero et al. 2019; Classen et al. 2020). Plant–pollinator networks at higher elevation can be more nested (Classen et al. 2020), but some studies find no relationship between elevation and nestedness (Cuartas-Hernández and Medel 2015) or find the opposite pattern (Ramos-Jiliberto et al. 2010). Variations in plant and pollinator diversity and community-level interaction networks have been largely attributed to environmental filtering effects (Ohler et al. 2020), but only rarely have they been linked to each other (Hoiss et al. 2012; Junker et al. 2019).

It is suggested that plant and pollinator diversity influence the general patterns of plant–pollinator interactions (i.e., network structure) (Junker et al. 2015). At the community level, the relationship between (functional) diversity and network structure in plant–pollinator systems has received little attention (but see Chamberlain et al. 2014; Junker et al. 2015; Souza et al. 2018). However, the research examining the link between overall community diversity and network level plant–pollinator interactions (i.e., macrostructure) suggests a negative relationship between diversity and interaction specialization; communities with greater diversity (species number and phylogenetic diversity) exhibit interaction networks characterized by lower measures of specialization (Chamberlain et al. 2014). At the species level, plant–pollinator studies have largely mirrored this pattern, showing that intraspecies *functional* diversity mediates patterns of plant–pollinator interactions (i.e., microstructure) (Junker et al. 2013, 2015, 2019); generally, greater intraspecies diversity mediates lower interaction specialization likely because greater intraspecific trait diversity increases the breadth of compatible phenotypes of the interaction partners. Thus, it is suggested that functional traits, and in particular the diversity in functional traits, shape the structure of the plant–pollinator network (Poisot et al. 2015; Valdovinos 2019).

Functional traits mediate the micro-structure of plant–pollinator interactions (Fontaine et al. 2006; Stang et al. 2006; Junker et al. 2013), which is the presence/absence and frequency of interactions between a plant and a pollinator species (Junker et al. 2010). Consequently, levels of community functional diversity (i.e., the variation of functional traits in a community) may strongly affect the structure of plant–pollinator interaction networks (Junker et al. 2015; Olito and Fox 2015). For example, high levels of functional trait diversity could give rise to higher plant–pollinator complementary specialization (H_2_’) because a larger variation in floral traits will allow a higher diversity of flower visitors to reach floral resources; in other words, a high floral functional diversity provides a larger fundamental niche space for their animal partners (Dehling et al. 2014; Junker et al. 2019). Larger niche spaces allow finer resource partitioning between consumers (Blüthgen and Klein 2011), particularly as individual species present traits that deviate from the community mean (Coux et al. 2016; Rumeu et al. 2018). Evidence for this relationship has been equivocal; simulations with empirically derived data indicated a positive correlation between functional diversity and complementary specialization (Junker et al. 2015), but this relationship has yet to be supported with field data (Souza et al. 2018). Alternatively, high levels of functional diversity could give rise to lower network-level specialization because larger niche spaces could also support species that are hyper-generalist (i.e., species that can exploit larger traits spaces) and/or a higher number of generalist species (Blüthgen and Klein 2011). This relationship has some support, too. For example, a study showed that generalist flower visitors occupy larger floral trait spaces (Kuppler et al. 2017); in another study, high functional diversity was shown to be positively correlated with nestedness (Chamberlain et al. 2014).

In this study, we observed interactions between flowering plants and their pollinators, and we determined the functional diversity of both groups in 24 communities along an elevational gradient in the Austrian Alps. The aim of this study is to better understand how elevation changes community composition in terms of species diversity, phylogenetic diversity, functional diversity, and plant–pollinator network structure. In turn, changes in community composition may elucidate how species, functional diversity and plant–pollinator interaction networks are related. First, we explored how plant and pollinator diversity change with elevation. We predicted that as elevation increases, species diversity (i.e., Hill diversity), phylogenetic diversity and functional diversity of plants and pollinators would decline in a concerted manner. Second, we investigated whether the patterns of plant–pollinator interactions, quantified as pollination network structure indices, also change along the elevational gradient. Specifically, we assessed changes in three specialization network indices that represent patterns of interactions relevant to pollination services. We predicted that as elevation increases, specialization would decline (i.e., lower complementary specialization and modularity; higher nestedness). We also assessed how network structure is connected to overall plant and pollinator functional diversity. For plants, we also assessed the relationship between network structure and the diversity of various subsets of floral traits with the goal of empirically discerning which floral traits are of functional relevance for network structure. By examining the relationship between plant–pollinator community functional diversity and interaction networks, we aim to find mechanistic link between the functional diversity of plants and pollinators and the functioning of alpine pollination system along an elevational gradient. These data may also allow for (careful) conclusions about how climate change may affect plant–pollinator networks.

## Methods

### Study system

We studied communities at eight elevations in the Austrian Alps following the methodological procedure described in Junker et al. (2019). Briefly, the communities were located in the National Park Hohe Tauern (Land Salzburg, Austria), along an elevational gradient from 1179 to 2597 m.a.s.l. Sites were selected based on accessibility and to evenly cover the elevational gradient. In each site, three 30-m transects were established and visited once per month. Sampling occurred from May to September in 2016 and 2017. Two 2 × 2 m plots per transect were established to conduct vegetation surveys (*n* = 121) following a modified abundance-dominance scale based on Braun-Blanquet (1964) to assess plant species abundance (cover per species in percent). For statistical analysis we used the recorded values of plant cover in percent and substituted + and *r* with 0.5 and 0.1, respectively. The number of inflorescences were recorded for all floral species and pooled to estimate total floral abundance per transect.

### Flower–visitor interactions

Flower–visitor interactions were recorded on sunny days between 8:45 and 18:00 h. We conducted a total of 288 h of observations, which occurred over 76 days. Each transect was sampled by walking slowly along the transect and collecting all insects that were observed to come in contact with a flower. For species with long nectar tubes or concealed floral parts, we checked for insects hidden inside the flowers. On each transect, we recorded flower–visitor interactions on 3 days per month throughout the field season for a period of 24 min each (i.e. 720 min observation time per transect), following a randomized order. Plant–pollinator observations were conducted by up to four persons simultaneously. All collected insects were then stored in a freezer for identification, trait measurements, and for counting pollen on the insect bodies. We were unable to identify all insects to species or genus, hence we calculated pollinator diversity at the morphospecies level for insects (see Supplemental Table S1 for complete list of insects observed). In total, we observed *n* = 9369 interactions between plants and flower–visitors, however, only *n* = 5735 were determined to be between plants and pollinators. We chose to consider only interactions where the floral visitor carried pollen, as not all floral visitors move pollen from flower to flower. The number of pollinators of each species collected were recorded and pooled to estimate total pollinator abundance per transect.

### Pollinator and plant morphology

For each insect morphospecies, we measured body length, head diameter, and proboscis length (fully extended) using calipers for *n* = 7–10 individuals. Body length was measured as the distance between the tip of the head and the tip of the abdomen, not including appendages. Additionally, we estimated the amount of pollen attached onto insects’ head, thorax, legs and abdomen to differentiate between pollinating and non-pollinating floral visitors. We assigned a score for each of the insects’ body parts, based on the number of pollen grains attached: 0 (no pollen), 1 (1–4 pollen grains), 2 (5–10 pollen grains), 3 (11–100 pollen grains), 4 (100 + pollen grains). Insect taxa with an average pollen load score below 1 were considered “non-pollinating” floral visitors and were removed from the analyses.

For plant taxa, we measured 19 floral traits that characterize flower morphology, size, color, nectar tube morphology, reproductive organ position, and inflorescence size and number. For flower morphology, we measured flower diameter, floral depth, petal width, petal length, and flower inclination. Flower inclination was obtained by measuring the angle of direction of the flower opening, where downward facing flowers would have angles measuring under 90º and upward facing flowers would have angles between 90° and 180°. For nectar tube morphology, we measured nectar tube width (diameter) and depth (distance between the constricting part of the flower and the bottom of the nectar tube). For open flowers and bowl-shaped flowers, nectar tube width was measured as the diameter of the flower and the nectar tube depth was zero. For reproductive organ position (i.e., anthers and stigma), we measured length and position. Anthers and stigma positions were measured as the distance between the anthers/stigma and constricting part of the flowers, with positive values indicating anthers/stigma that protrude above the floral opening and negative values indicating anthers/stigma hidden in the floral tubes. For inflorescence-level floral traits, we measured display size, number of inflorescences, number of flowers per inflorescence, and plant height. Display size was measured as the length of the widest dimension of the inflorescence.

To measure inflorescence color, seven or ten floral units in anthesis per species (depending on abundance) were collected in the field. Samples were placed in plastic bags containing a moistened paper towel and stored in a cooler box until they were transported to the laboratory within a few hours. In the laboratory, the samples were stored in a refrigerator (4 °C) until color measurements were conducted on the same day. For spectrometric measurements, a JAZ-S spectrometer equipped with a JAZ-PX pulsed xenon lamp (Ocean Optics, Ostfildern, Germany) as light source was used. For each plant, flower color spectra were measured between 300 and 700 nm. If a flower displayed two or more colors, the base color according to the biolFlor database (Kühn et al. 2004) was measured. Reflection was measured at a 45° angle along the longitudinal axis of the petal with the light source facing toward the basal part. Based on spectra and using the *pavo* package ver. 2.0.0 (Maia et al. 2019), we obtained four color component measures based on short, medium, and long wavelength bee vision receptors, and luminance. Note that we chose to only use bee vision receptors to assess flower color diversity as a proxy for overall flower color diversity. The use of additional color vision systems would have inflated the data analysis. Accordingly, interpretation of results based on color need to be cautious.

### Community diversity and network structure

To understand how community diversity changes along the elevational gradient in our study, we employed indices of taxonomic, phylogenetic, and functional diversity for flowering plants and pollinators. To measure community taxonomic diversity, we calculated species diversity (Hill diversity) and phylogenetic diversity (Faith’s PD) for both pollinators and plants using the *iNEXT* (Hsieh et al. 2016), *ape* (Paradis and Schliep 2019), *pez* (Pearse et al. 2015), and *picante* (Kembel et al. 2010) R packages. To quantify Hill diversity, we defined *q* = *1* to obtain a Hill diversity index that does not over emphasize either rare or common species in a manner similar to the often used Shannon diversity index (Roswell et al. 2021). This variant of Hill diversity is referred to as Hill–Shannon diversity.

To measure the functional diversity per community (i.e., transect) for plants and pollinators, we calculated unweighted and weighted *n*-dimensional hypervolumes using the primary function in the *dynRB* package (Junker et al. 2016). Here, we employ a framework that utilizes Hutchinsonian *n*-dimensional hypervolumes (Junker et al. 2016). Under this framework, each functional trait in a community can be represented by one dimension in a hypervolume, with the range of trait values defining the trait space breadth along that dimension. By employing dynamic range boxes, it is possible to examine functional diversity in a qualitative (unweighted trait space) and quantitative (weighted trait space) manner (see Junker et al. 2016 for a detailed description of dynamic range boxes). Both perspectives on functional diversity provide deviating information on the availability of floral niches: The weighted trait space considers floral abundances per species and thus reflect quantity of flower resources per species. The unweighted trait space informs about the number of niches (quality) but neglects the quantitative aspect. *dynRB*’s main function allows for the calculation of both unweighted and weighted hypervolume trait spaces and its non-parametric approach avoids any assumptions of data normality and is robust against outliers. For additional information on the calculation of weighted hypervolumes see Kuppler et al. (2017). Hypervolume sizes were quantified using the “gmean” (geometric mean) aggregation method to allow for direct comparisons between hypervolumes calculated using unequal numbers of trait dimensions.

We calculated hypervolumes that included all traits measured, as well hypervolume variations using only subsets of traits based on specific functions. Hereafter, when referring to unweighted and weighted trait spaces, we are explicitly referring to functional diversity measured as the unweighted and weighted hypervolumes, respectively. For plants, we calculated unweighted and weighted hypervolumes using six sets of floral traits and for each set of traits. The floral traits subsets included all floral traits, display (except color), morphology (i.e., traits relevant to accessibility for pollinators), nectar, pollen (location), and color traits (see Supplemental Fig. S2 and Table S2 for all correlations and a list of specific traits included in each subset). For pollinators, we calculated one weighted and unweighted hypervolume using the three measured morphological traits.

To measure changes in plant–pollinator interactions along the elevation gradient, we examined changes in three topology indices of the plant–pollinator networks. Plant–pollinator networks were compiled as matrices where animals and plants are represented as columns and rows in a matrix, respectively, and each cell value represents the number of interactions observed. Based on these matrices, we calculated the H_2_’ (complementary specialization), modularity Q and weighted NODF (weighted nestedness metric based on overlap and fill) using functions within the *bipartite* R package (Dormann et al. 2008). H_2_’ quantifies the degree of mutual specialization between two organisms for the entire network (Blüthgen et al. 2006). Modularity refers to the tendency of clusters of species to form within the network, where species are strongly interlinked with others within the cluster and weakly interlinked with species outside the cluster (Olesen et al. 2007). Nestedness (weighted NODF) depicts the tendency of specialists in the network to interact with generalists (Bastolla et al. 2009; Almeida-Neto and Ulrich 2011).

### Statistical analysis

We assessed correlations between diversity and elevation, network structure and elevation, and network structure and functional diversity. To explore these relationships, we first calculated Pearson’s product moment correlation coefficients for all pairs of diversity indices, network structure indices and elevation using the corrplot R package (Wei and Simko 2017; Supplemental Fig. S2). Correlations were considered statistically significant when *p* < 0.05. Upon a visual inspection of the data, it appeared that the relationships between the various diversity indices and elevation did not always follow a linear relationship. Hence, we build three models: one model included only the true elevation (i.e. linear model), one included a squared term for elevation (i.e., unimodal model), and one included a cubic term for elevation (i.e., bimodal model). We then compared between the three competing models using Akaike Information Criterion (AIC) values and parsimony; a more complex model was deemed better only if its AIC value was 2 units lower than a simpler one.

Because network indices were correlated with multiple indices of taxonomic and functional diversity (see Results section and Supplemental Fig. S2), we proceeded with a post hoc analysis to determine the relationships between diversity measures and network structure using structural equation models (SEMs) with the primary function in the *piecewiseSEM* R package (Lefcheck 2016). Briefly, structural equation modeling is a framework that evaluates cause–effect relationships in complex multivariate models (Grace et al. 2015). Specifically, SEMs enable the simultaneous assessment of multiple relationships to discern direct and indirect effects. In this study, we specified separate models for each network index and assessed the effects of taxonomic and functional diversity in plants and pollinators, separately. In the SEMs, we also included floral and pollinator abundances, to account for the large role that abundance plays in network structure (Vázquez et al. 2009). In total, we built six distinct predicted SEMs (3 network indices × 2 taxonomic groups). However, our approach comes with some caveats, as we largely ignore the obvious relationships between floral and pollinator diversity. This choice reflects a limitation due to a small sample size to estimated parameter ratio (*d* = *4.8*). Grace et al. (2015) recommends a sample size to estimated parameter ratio between 5 and 20, as lower sample size to parameter ratios can result in poor parameter estimation and model evaluation (Deng et al. 2018). For a description of the predicted SEMs see Supplemental Fig. S3.

Following the specification of the predicted SEMs we proceeded with model selection. Model fit was examined in each SEM by performing Shipley’s d-separation tests (Shipley 2013), which test whether any missing paths should be included for a better fit. From the d-separation tests, a Fisher’s C test statistic is calculated and used as a measure of overall model fit. Fisher’s C statistic is then used to calculate an Akaike Information Criterion (AIC) value for model comparisons (Shipley 2013). To find the best possible models, we simplified models for comparison by removing the least significant path (largest *p* value) in each model and comparing the AIC value yielded by the unreduced and reduced model. An unreduced model was deemed better only if its AIC value was 2 units lower than the reduced model. In cases where two paths had nearly identical *p* values, we declared two distinct reduced models by dropping only one of the alternative paths at a time, and then compared the two reduced models to the unreduced one.

All statistical analyses were performed using the statistical computing software R ver. 4.1.0 (R Core Team 2021) and all plots were created using the graphics (base), ggplot2 R package (Wickham 2016).

## Results

### Diversity and elevation

**Fig. 1:**
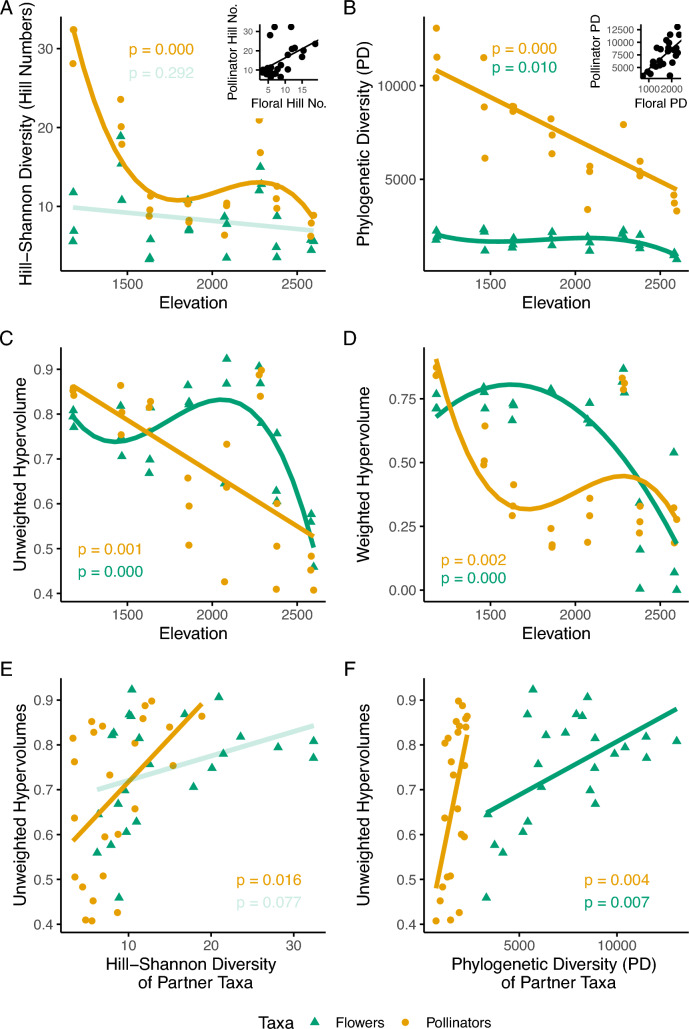
Relationships between community diversity and elevation. Panels **A** and **B** depict the relationships between elevation and two measures of taxonomic diversity, Hill–Shannon diversity and phylogenetic diversity, respectively. Panels **C** and **D** depict the relationships between elevation and unweighted and weighted hypervolumes, respectively. Panels **E** and **F** depict the relationship between unweighted trait spaces and taxonomic diversity of their partner taxa, meaning that when we refer for example to the unweighted hypervolumes for the pollinator community, the predictor measured is the taxonomic diversity of the plant community. Green lines (and triangles) represent the trends and data for measures of plant diversity and the orange lines (and circles) represent the trends and data for measures of pollinator diversity. Solid lines depict statistically significant relationships (p < 0.05) and transparent lines depict nonsignificant relationships

Most components of plant and pollinator diversity correlated with elevation (Table 1). As elevation increased, floral Hill–Shannon diversity decreased but this relationship was not statistically significant (Fig. 1A). On the other hand, floral phylogenetic diversity exhibited a fluctuating relationship with elevation, which was defined by a drop off at the highest elevation (Fig. 1B). Floral unweighted trait space (i.e., unweighted hypervolumes) exhibited a similar relationship with elevation, peaking in communities at middle elevations, with communities at both elevational extremes occupying smaller trait spaces; communities at the highest elevations occupied the smallest trait space (Fig. 1C). Floral weighted trait space (i.e., weighted hypervolumes) appeared to be similar at most elevations but declined sharply at the highest elevations (Fig. 1D).

**Table 1:** Summary statistics for correlations of diversity indices with elevation

Diversity index	Plants				Pollinators			
	*F*	*p*	*Adjusted r*^*2*^	*Degree*	*F*	*p*	*Adjusted r*^*2*^	*Degree*
Phylogenetic diversity	**4.971**	**0.010**	**0.341**	**3**	**32.316**	**0.000**	**0.577**	**1**
Hill–Shannon diversity	1.167	0.292	0.007	1	**22.500**	**0.000**	**0.737**	**3**
Unweighted trait space (unweighted hypervolumes)	**10.946**	**0.000**	**0.565**	**3**	**14.985**	**0.001**	**0.378**	**1**
Weighted trait space (weighted hypervolumes)	**13.336**	**0.000**	**0.518**	**2**	**7.435**	**0.002**	**0.456**	**3**

Boldfaced numbers depict statistically significant relationships (*p* < 0.05)

Note that we report adjusted *r*^2^ values to account for multiple predictors in the linear models. Degree columns denote models with quadratic and cubic terms, respectively

As elevation increased, all pollinator diversity measures declined. Pollinator Hill–Shannon diversity and weighted trait spaces declined sharply at middle elevations, remaining somewhat constant through higher elevations, except for a high diversity community at one elevational step (i.e., ~ 2300 m.a.s.l.; Fig. 1A and D). Pollinator phylogenetic diversity and unweighted traits spaces both appeared to decrease linearly as elevation increased (Fig. 1B, C).

As suggested by the overall declines in plant and pollinator diversity along the elevational gradient, we detected strong correlations between the taxonomic diversity metrics of plants and their pollinators (Hill–Shannon diversity: *t*_*22*_ = 2.71, *p* = 0.013, *r*^2^ = 0.25; phylogenetic diversity: *t*_*22*_ = 4.242, *p* < 0.001, *r*^2^ = 0.45; Fig. 1A, B insets). We also detected strong correlations between the functional diversity metrics of plant and their pollinators (unweighted hypervolumes: *t*_*22*_ = 3.90, *p* < 0.001, *r*^*2*^ = 0.41; weighted hypervolumes: *t*_*22*_ = 2.29, *p* = 0.03, *r*^*2*^ = 0.19). Additionally, we found that pollinator unweighted and weighted trait space sizes were positively correlated with both taxonomic diversity metrics of the flowering plants in the community, while plant unweighted and weighted trait spaces were only significantly correlated with the phylogenetic diversity of the pollinators (Fig. 1E, F).

### Pollination network structure and diversity

**Fig. 2:**
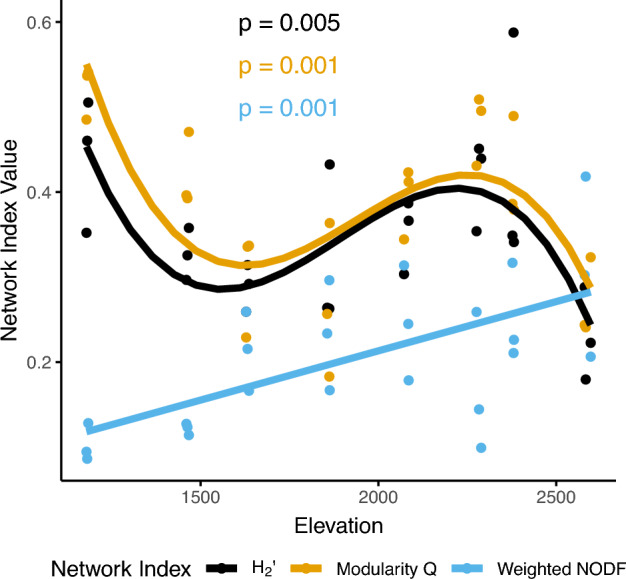
Relationships between network indices and elevation. Note that weighted NODF values range from 0 to 100, but we have scaled the observed values, dividing them by 100, to compare its change with that other network indices. All relationships are statistically significant at *p* < 0.05. H_2_’ quantifies the degree of mutual specialization between two organisms for the entire network. Modularity refers to the tendency of clusters of species to form within the network, where species are strongly interlinked with others within the cluster and weakly interlinked with species outside the cluster. Nestedness (weighted NODF) depicts the tendency of specialists in the network to interact with generalists

We found significant relationships between elevation and all three observed network structural properties (Fig. 2, Supplemental Table S4). Complementary specialization (H_2_’) and modularity displayed similar fluctuating relationships with elevation, while nestedness (weighted NODF) increased linearly along the elevational gradient.

**Fig. 3:**
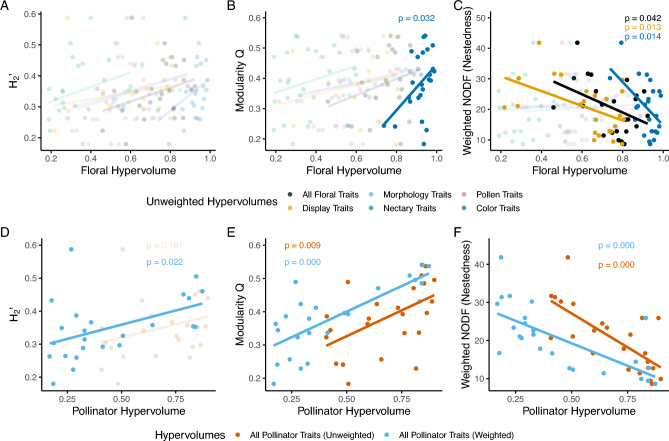
Plant–pollinator network structure indices in relation to floral unweighted traits spaces as measured by the size of unweighted hypervolumes (Panels **A**–**C**). Plant–pollinator network structure indices in relation to pollinator weighted trait spaces as measured by the size of weighted hypervolumes (Panels **D**–**F**). Solid lines depict statistically significant relationships (*p* < 0.05) and transparent lines and points depict nonsignificant relationships

Network indices were not consistently correlated with floral functional diversity (Fig. 3A–C, Table 2, Supplemental Table S4). We did not find complementary specialization (H_2_’) and modularity to be correlated with floral trait spaces (Fig. 3A, B), except for a correlation between modularity and the unweighted, color-only traits hypervolumes. On the other hand, nestedness was negatively correlated with floral unweighted and weighted trait spaces (Fig. 3C, Supplemental Table S4). When examining whether certain subsets of floral traits may mediate nestedness, we found that floral display (unweighted and weighted) and color traits hypervolumes (unweighted only) were negatively correlated with nestedness.

**Table 2:** Summary statistics for relationships between network indices and unweighted floral trait hypervolumes

Hypervolume variant (unweighted)	H_2_'			Modularity Q			Weighted NODF		
	*t*	*p*	*r*^*2*^	*t*	*p*	*r*^*2*^	*t*	*p*	*r*^*2*^
All floral traits	1.381	0.181	0.080	1.541	0.138	0.097	**− 2.158**	**0.042**	**0.175**
Display (no color)	1.000	0.328	0.044	1.438	0.164	0.086	**− 2.713**	**0.013**	**0.251**
Morphology	1.444	0.163	0.087	0.737	0.469	0.024	0.296	0.770	0.004
Nectary	1.430	0.167	0.085	1.218	0.236	0.063	0.345	0.733	0.005
Pollen	1.342	0.193	0.076	0.987	0.335	0.042	− 0.177	0.861	0.001
Color	1.679	0.107	0.114	**2.288**	**0.032**	**0.192**	**− 2.671**	**0.014**	**0.245**

Boldfaced numbers depict statistically significant relationships (*p* < 0.05)

Conversely, network indices almost always correlated with pollinator functional diversity (Fig. 3D–F, Table 3). Modularity and nestedness were both correlated with pollinator unweighted trait spaces, while complementary specialization (H_2_’) was not, and all three network indices were correlated with pollinator weighted trait spaces.

**Table 3:** Summary statistics for relationships between networks indices and pollinator trait hypervolumes

Hypervolume variant	H_2_'			Modularity Q			Weighted NODF		
	*t*	*p*	*r*^*2*^	*t*	*p*	*r*^*2*^	*t*	*p*	*r*^*2*^
All pollinator traits (unweighted)	1.451	0.161	0.087	**2.887**	**0.009**	**0.275**	**-4.869**	**0**	**0.519**
All pollinator traits (weighted)	**2.459**	**0.022**	**0.216**	**5.517**	**0.000**	**0.580**	**-4.568**	**0**	**0.487**

Boldfaced numbers depict statistically significant relationships (*p* < 0.05)

### Structural equation models

**Fig. 4:**
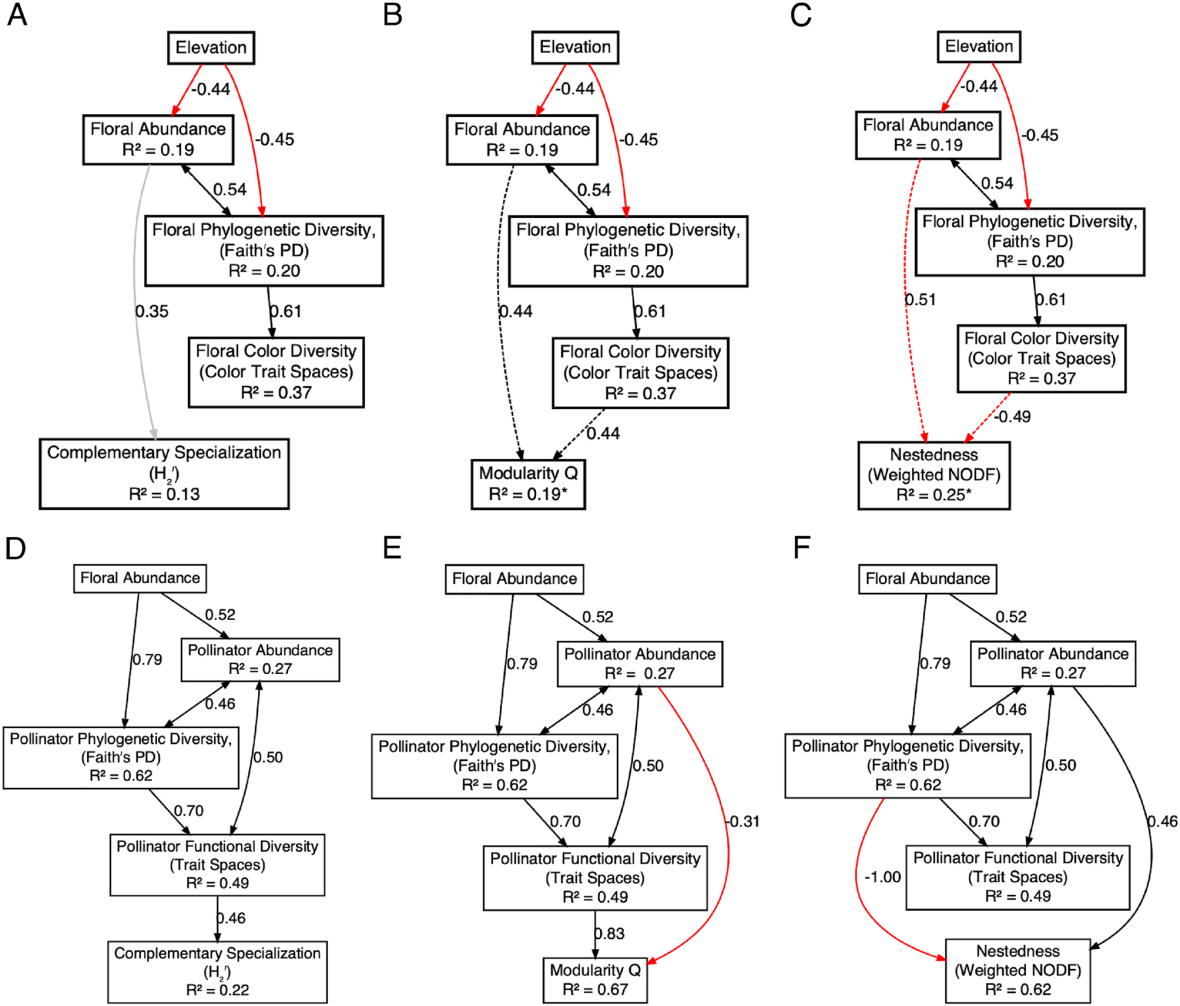
Structural equation models (SEM’s) describing the relationships between elevation, plant (Panels **A–C**) and pollinator diversity (Panels **D–F**), and network structure. Solid black and red lines depict positive and negative relationships, respectively, that are statistically significant (*p* < 0.05). The gray line depicts a marginally significant, positive relationship (0.10 > *p* > 0.10). Dashed lines depict two paths with nearly equal support in models with only one of the paths specified (i.e., less than 2 AIC units in difference between competing models). Asterisks denote individual *r*^*2*^ values obtained by averaging the *r*^*2*^ from the two competing SEMs

All three floral SEMs indicate that elevation had a negative impact in floral abundance and flowering plant phylogenetic diversity (Fig. 4A–C). In turn, floral functional diversity (i.e., color trait spaces) increased with plant phylogenetic diversity. All three floral SEMs failed to yield clear associations between floral diversity and network structure. For instance, only floral abundance appears to influence complementary specialization (H_2_’), but this relationship is only marginally significant (0.10 > *p* > 0.05; Fig. 4A). Meanwhile, the SEMs failed to clearly indicate that floral diversity mediates modularity or nestedness, as competing models with either floral abundance or functional diversity as network structure mediators have nearly identical support (Fig. 4B, C) and the proportion of variation explained in these models is relatively low (i.e., low *r*^*2*^ values).

All three pollinator SEMs indicate that floral abundance had a positive effect on pollinator abundance and pollinator phylogenetic diversity (Fig. 4D–F). In turn, pollinator functional diversity (i.e., trait spaces) increased with pollinator phylogenetic diversity. Complementary specialization (H_2_’) appeared to be mediated solely by pollinator functional diversity (*r*^*2*^ = 0.22; Fig. 4D). Modularity appeared to be mediated by pollinator abundance (negative correlation) and functional diversity, with these two factors explaining a large proportion of the variance in modularity (*r*^*2*^ = 0.67; Fig. 4E). We also observed significant effects from pollinator abundance (positive) and phylogenetic diversity (negative) on nestedness (weighted NODF; Fig. 4F). However, it is important to note that the high standardized path coefficient between pollinator phylogenetic diversity and nestedness indicates a possible collinearity issue, although the Variance Inflation Factor (*VIF* = 1.73) and the Condition Index (*CI* = 8.15) are only very slightly elevated. While this hinders a clear interpretation of the effects of pollinator abundance and phylogenetic diversity, these two factors explain a relatively high proportion of the variation in network nestedness (*r*^*2*^ = 0.62).

## Discussion

Understanding the relationship between diversity and ecosystem processes remains a primary goal in pollination studies (Kaiser-Bunbury et al. 2017; Woodcock et al. 2019). By employing a functional trait framework (McGill et al. 2006), it is possible to relate plant and pollinator species traits with plant–pollinator network micro-structure (Stang et al. 2006; Junker et al. 2013, 2019; Kaiser-Bunbury et al. 2014). However, to our knowledge fewer studies have attempted to relate overall plant and pollinator community functional diversity with the community level patterns of plant–pollinator interaction networks (Chamberlain et al. 2014; Junker et al. 2015; Souza et al. 2018). In our study, we found strong relationships between elevation, species diversity, functional diversity, and network structure. Specifically, we found that plant and pollinator communities that are more taxonomically and functionally diverse exhibited plant–pollinator interaction networks that are more specialized, more modular, and less nested.

In this study, we observed community changes along an elevational gradient to investigate how species diversity, functional diversity and plant–pollinator network structure are related. Species diversity and phylogenetic diversity for plants and pollinators both declined with elevation, following a well-established trend in alpine community studies (Sundqvist et al. 2013; Minachilis et al. 2023). Elevation also had an impact on floral functional diversity, and as shown by Junker and Larue-Kontic (2018), flowering communities at mid-elevations exhibited the highest functional diversity. As elevation increased, pollinator functional diversity also declined. As expected, elevation also influenced network structure; as elevation increases complementary specialization and modularity decline in an oscillating manner, while nestedness increases linearly. These results corroborate previous works that showed a decline in the specialization and modularity at higher elevations in various alpine ecosystems (Hoiss et al. 2015; Lara-Romero et al. 2019; Classen et al. 2020). For nestedness we found a positive relationship with elevation, which is confirmed by some but not all studies investigating the same relationship (Ramos-Jiliberto et al. 2010; Cuartas-Hernández and Medel 2015; Classen et al. 2020).

Plant–pollinator network structure was correlated with both floral and pollinator functional diversity, but we found that network structure was slightly more likely to be correlated with unweighted trait spaces in flowering plants, namely unweighted color traits spaces (Table 2; Table S5). We also found that network structure measures are almost as likely to be correlated with weighted or unweighted trait spaces in pollinators. The key difference between unweighted and weighted measures of functional diversity is the contribution of rare species in the quantification of the trait spaces (see Supplemental Fig. S1). The quantification of weighted traits spaces puts less weight on rare species, while these species are considered on equal terms with more common species in the unweighted trait spaces. Thus, the relationship between network structure and these measures of functional diversity may point to two distinct mechanisms by which floral and pollinator traits mediate plant–pollinator interactions. On the one hand, network structure is affected (weakly) by rare floral color phenotypes, while on the other, rare pollinator phenotypes do not seem to play a significant role.

We found that pollinator diversity was a better predictor of network structure than plant diversity. The diversity of all pollinator traits was correlated with all three measures of network structure, however, the SEM’s evaluating the effect of diversity measure on network structures suggest complementary specialization and modularity are driven by functional diversity, while nestedness is driven primarily by phylogenetic diversity. We speculate that greater pollinator functional diversity mediates greater specialization due to the larger variation in traits often associated with trait-matching in plant–pollinator interactions, such as proboscis length and body size (Stang et al. 2006, 2009), as great variation allows for finer resource partitioning between competing pollinators (Blüthgen and Klein 2011). The relationship between morphological pollinator traits and network structure may suggest that greater diversity in the corresponding floral traits (i.e., nectary depth, floral diameter) would also mediate specialization and modularity. However, this does not appear to be the case as our results fail to clearly indicate that greater floral functional trait diversity leads to greater complementary specialization or modularity. Although, unweighted color trait spaces sizes do correlate with modularity, this may be a spurious correlation. On the other hand, our study does suggest that functional plant diversity may reduce network nestedness, but the evidence is not conclusive given that SEMs with and without a causal path between functional diversity and nestedness do not differ in their level of support.

Altogether, the results presented here provide some insights into the mechanisms underlying plant–pollinator network structure. First, it is apparent that the functional diversity of each taxon differs in their relationship with network structure. Morphological diversity traits in pollinators were able to explain a relatively large proportion of the variation in community level network structure, despite missing other important functional pollinator traits, such as hairiness, behavioral patterns and phenology (Goulnik et al. 2020; Murúa 2020). On the contrary, morphological diversity traits in plants, and their abundance, exhibited low explanatory power. This is interesting given the large number of floral traits measured in our study and raises questions about how the diversity of other floral traits may impact network structure. For example, we did not assess nutritional content and secondary chemistry in floral rewards, floral scent emissions or phenology, all of which have independently been shown to mediate plant–pollinator interactions (Hanley et al. 2008; Olesen et al. 2008; Adler et al. 2012; Larue et al. 2016; Kantsa et al. 2018). Future studies assessing the effects of functional diversity should incorporate these traits. Second, it is also worth noting that although the results presented in this study corroborate our initial prediction, they depict a relationship between diversity and network structure that differs from a similar plant–pollinator study by Chamberlain et al (2014), which was carried over a larger spatial scale and that spanned several community types. We presume that the differences between our results and those in Chamberlain et al (2014) may be partially explained by the rather large variation in the community types sampled in their study, an important sampling difference. These differences point to the need for greater sampling standardization and replication of plant–pollinator network studies (Pellissier et al. 2018).

The dynamics of plant–pollinator communities in alpine regions are important to consider in the face of imminent climate change. Alpine ecosystems are thought to be particularly susceptible to the effects of climate change (Seddon et al. 2016) and they presently host a disproportionate fraction of plant and pollinator diversity (for example Minachilis et al. 2023). As climate change progresses, the environmental conditions will inevitably shift and changes in those communities will ensue. What exactly those changes will be and how they will affect interactions between plants and pollinators is difficult to predict. On one hand, plants and pollinators already present at higher elevations will be increasingly likely to go extinct as they lose habitable land area (Dirnböck et al. 2011). But an important question to ask is how the relatively high nestedness and low modularity/specialization of communities at higher elevations will affect extinction rates. Research suggests that nestedness can mitigate extinction rates, as the extinction of the most at-risk species may not necessarily induce the extinctions of their less specialized partners (Memmott et al. 2004). On the other hand, research also suggests that modularity and complementary specialization can mitigate extinction rates by buffering the network from the spread of extinctions (Stouffer and Bascompte 2011; Sonne et al. 2022). A key question for future research is how the dynamics of taxonomic and functional diversity losses in plant and pollinator communities, resulting from environmental changes and habitat loss, will interact with the purported extinction tolerance of nested, modular, and specialized networks. Additionally, mountainous regions could play a major role in biodiversity conservation. Alpine ecosystems could serve as refugia by allowing for some species whose environmental needs are not being in the lowlands to “escape” onto higher elevations that present more favorable conditions (Meng et al. 2019). However, determining the existence of this refugia scenario is challenging, as it depends on whether species, both common and rare, can move to higher elevations, if their specialized partners can follow suit, and whether further human-induced degradation of alpine ecosystems persists. This potential scenario underscores the urgency of preserving pristine alpine ecosystems.

## Conclusions

Our research confirms that alpine ecosystems present unique opportunities to test hypotheses regarding the effects of climate change on the diversity of plant and pollinator communities, impacting their interactions by utilizing alpine communities in space-for-time studies (Sundqvist et al. 2013). Our study suggests that greater plant and pollinator diversity is linked to plant–pollinator network structural properties, such as complementary specialization, modularity, and nestedness. These relationships shown here provide some reference points for understanding how interactions could be reshaped under future climate scenarios. But the study of plant–pollinator diversity and interaction networks will require long-term research projects that track how those communities and the interactions between their members change under climatic conditions. Additionally, more research is needed to test whether high diversity in plant–pollinator systems and species actually improves the quality of pollination services and what this will mean for rapidly changing communities, such as alpine ecosystems. In conclusion, understanding the interplay between plant and pollinator functional diversity, network structure, and the influence of climate change is crucial for comprehending and managing the future of plant–pollinator interactions under climate change scenarios.

## Supplementary Information

Below is the link to the electronic supplementary material.

Supplementary file1 (DOCX 381 kb)
