# Supplementary material for: Floral and pollinator functional diversity mediate network structure along an elevational gradient
Source: Alp Bot. 2024 Mar 16;134(2):193–206. doi: 10.1007/s00035-024-00308-w (PMC11607024; doi:10.1007/s00035-024-00308-w)
Supplement: Supplementary file 1 — Supplementary file1 (DOCX 381 kb) [file 35_2024_308_MOESM1_ESM.docx]

SUPPLEMENTARY INFORMATION

FLORAL AND POLLINATOR FUNCTIONAL DIVERSITY MEDIATE NETWORK STRUCTURE ALONG AN ELEVATIONAL GRADIENT

LUIS A. AGUIRRE^1,2,3^, ROBERT R. JUNKER^1,4*^

^1^*Department of Biosciences, University of Salzburg, Austria*

^2^*Department of Biology, University of Massachusetts, Amherst, MA, USA*

^3^*Graduate Program in Organismic and Evolutionary Biology, University of Massachusetts, Amherst, MA, USA*

^4^*Evolutionary Ecology of Plants, Department of Biology, University of Marburg, Marburg, Germany*

*Corresponding Author: robert.junker@uni-marburg.de

**Table S1** List of flower-visitors collected in all 24 communities and elevation at which they are found. In the Pollen column, “X” marks identify which species were found to carry pollen on their bodies.

| **Taxonomic Level** | | | | **Elevation** | **Pollen** |
| --- | --- | --- | --- | --- | --- |
| **Order** | **Family** | **Genus** | **Species** |  |  |
| Acari | Acari | *Acari* | *Acari sp.* | 1860 |  |
| Araneae | Lycosidae | *Lycosidae* | *Lycosidae sp.* | 1632, 2379 |  |
| Araneae | Pisauridae | *Pisauridae* | *Pisauridae sp.* | 2283 |  |
| Araneae | Salticidae | *Salticidae* | *Salticidae sp.* | 1463 |  |
| Araneae | Tetragnathidae | *Tetragnathidae* | *Tetragnathidae sp.* | 2587 |  |
| Araneae | Thomisidae | *Thomisidae* | *Thomisidae sp.* | 1181 |  |
| Coleoptera | Buprestidae | *Buprestidae* | *Buprestidae sp.* | 1632 | X |
| Coleoptera | Cantharidae | *Cantharidae* | *Cantharidae sp.* | 1181, 1463, 1632 | X |
| Coleoptera | Carabidae | *Carabidae* | *Carabidae sp.* | 2080 |  |
| Coleoptera | Cerambycidae | *Cerambycidae* | *Cerambycidae sp.* | 1181, 1463, 1860, 2080, 2379 | X |
| Coleoptera | Chrysomelidae | *Bruchidae* | *Bruchidae sp.* | 1463 |  |
| Coleoptera | Chrysomelidae | *Chrysomelidae* | *Chrysomelidae sp.* | 1181, 1463, 2080, 2587 |  |
| Coleoptera | Cleridae | *Cleridae* | *Cleridae sp.* | 1632 | X |
| Coleoptera | Coccinellidae | *Coccinellidae* | *Coccinellidae sp.* | 1181, 1463, 2587 |  |
| Coleoptera | Cryptophagidae | *Cryptophagidae* | *Cryptophagidae sp.* | 1860 | X |
| Coleoptera | Curculionidae | *Anoplus* | *Anoplus sp.* | 1181, 1632, 2283 | X |
| Coleoptera | Curculionidae | *Curculionidae* | *Curculionidae sp.* | 1463, 1860, 2080, 2283 | X |
| Coleoptera | Elateridae | *Elateridae* | *Elateridae sp.* | 1181, 1463, 1860, 2080, 2283, 2587 | X |
| Coleoptera | Melyridae | *Melyridae* | *Melyridae sp.* | 2283 | X |
| Coleoptera | Nitidulidae | *Nitidulidae* | *Nitidulidae sp.* | 1181, 1632 |  |
| Coleoptera | Oedemeridae | *Oedemeridae* | *Oedemeridae sp.* | 1463 | X |
| Coleoptera | Ptinidae | *Ptinidae* | *Ptinidae sp.* | 1463 |  |
| Coleoptera | Scarabaeidae | *Phyllopertha* | *Phyllopertha horticola* | 1181, 1632, 1860, 2080, 2283, 2379 | X |
| Coleoptera | Scarabaeidae | *Scarabaeidae* | *Scarabaeidae sp.* | 1181, 1463, 2587 |  |
| Coleoptera | Staphylinidae | *Anthomyzidae* | *Anthomyzidae sp.* | 1860 |  |
| Coleoptera | Staphylinidae | *Anthophagus* | *Anthophagus sp.* | 1860, 2080, 2283, 2587 |  |
| Coleoptera | Staphylinidae | *Eusphalerum* | *Eusphalerum sp.* | 1181, 1632, 1860, 2080, 2283, 2379, 2587 |  |
| Coleoptera | Staphylinidae | *Staphylinidae* | *Staphylinidae sp.* | 1632, 2080, 2283, 2379, 2587 |  |
| Collembola | Collembola | *Collembola* | *Collembola sp.* | 2587 |  |
| Collembola | Dicyrtomidae | *Dicyrtomidae* | *Dicyrtomidae sp.* | 2379 |  |
| Collembola | Entomobryidae | *Entomobryidae* | *Entomobryidae sp.* | 2080, 2587 |  |
| Collembola | Isotomidae | *Isotomidae* | *Isotomidae sp.* | 2283, 2587 |  |
| Collembola | Katiannidae | *Katiannidae* | *Katiannidae sp.* | 2080 |  |
| Collembola | Sminthuridae | *Sminthuridae* | *Sminthuridae sp.* | 2080, 2283 |  |
| Diptera | Agromyzidae | *Agromyzidae* | *Agromyzidae sp.* | 1181, 1463, 1632, 1860, 2080, 2283, 2379, 2587 |  |
| Diptera | Agromyzidae | *Hydrophoria* | *Hydrophoria diabata* | 1860 |  |
| Diptera | Anthomyiidae | *Adia* | *Adia cinerella* | 1632 | X |
| Diptera | Anthomyiidae | *Anthomyia* | *Anthomyia liturata* | 1463 |  |
| Diptera | Anthomyiidae | *Anthomyiidae* | *Anthomyiidae sp.* | 1632, 1860, 2379 |  |
| Diptera | Anthomyiidae | *Anthophagus* | *Anthophagus sp.* | 1860 |  |
| Diptera | Anthomyiidae | *Botanophila* | *Botanophila fugax* | 1463 | X |
| Diptera | Anthomyiidae | *Botanophila* | *Botanophila sp.* | 1181, 1463, 1632, 1860, 2080, 2283, 2379, 2587 | X |
| Diptera | Anthomyiidae | *Botanophila* | *Botanophila striolata* | 2379 | X |
| Diptera | Anthomyiidae | *Botanophila* | *Botanophila varicolor* | 1181, 1463, 1632, 1860, 2080, 2283, 2379, 2587 | X |
| Diptera | Anthomyiidae | *Chiastocheta* | *Chiastocheta trollii* | 1632 | X |
| Diptera | Anthomyiidae | *Delia* | *Delia nuda* | 2283, 2379 |  |
| Diptera | Anthomyiidae | *Delia* | *Delia sp.* | 2379 | X |
| Diptera | Anthomyiidae | *Egle* | *Egle inermis* | 2283 | X |
| Diptera | Anthomyiidae | *Heterostylodes* | *Heterostylodes nominabilis* | 1181 |  |
| Diptera | Anthomyiidae | *Hydrophoria* | *Hydrophoria diabata* | 1860 |  |
| Diptera | Anthomyiidae | *Hydrophoria* | *Hydrophoria lancifer* | 1632, 1860 | X |
| Diptera | Anthomyiidae | *Hydrophoria* | *Hydrophoria sp.* | 1463 | X |
| Diptera | Anthomyiidae | *Hylemya* | *Hylemya vagans* | 1463, 1632, 1860 |  |
| Diptera | Anthomyiidae | *Hylemya* | *Hylemya variata* | 1632, 1860 |  |
| Diptera | Anthomyiidae | *Pegoplata* | *Pegoplata aestiva* | 1181, 1463, 1632, 1860, 2080, 2283, 2379, 2587 | X |
| Diptera | Anthomyiidae | *Phorbia* | *Phorbia fumigata* | 1860, 2379 |  |
| Diptera | Anthomyiidae | *Anthomyiidae* 2 | *Anthomyiidae sp.* 2 | 1181, 1463, 1632, 1860, 2080, 2283, 2379, 2587 | X |
| Diptera | Anthomyiidae | *Delia* 2 | *Delia sp.* 2 | 1181 |  |
| Diptera | Anthomyzidae | *Anthomyzidae* | *Anthomyzidae sp.* | 1860, 2283 |  |
| Diptera | Asilidae | *Asilidae* | *Asilidae sp.* | 1181, 2587 |  |
| Diptera | Athericidae | *Athericidae* | *Athericidae sp.* | 1632 |  |
| Diptera | Bibionidae | *Bibionidae* | *Bibionidae sp.* | 1181, 1463, 1632, 1860, 2080, 2283 | X |
| Diptera | Bibionidae | *Dilophus* | *Dilophus sp.* | 1860 | X |
| Diptera | Calliphoridae | *Calliphoridae* | *Calliphoridae sp.* | 1181, 1463, 1632, 2080, 2283 | X |
| Diptera | Camillidae | *Camillidae* | *Camillidae sp.* | 2080 |  |
| Diptera | Carnidae | *Carnidae* | *Carnidae sp.* | 1181, 1860 |  |
| Diptera | Carnidae | *Meoneura* | *Meoneura sp.* | 1181 |  |
| Diptera | Cecidomyiidae | *Cecidomyiidae* | *Cecidomyiidae sp.* | 1181, 1860, 2080, 2283, 2379 |  |
| Diptera | Cecidomyiidae | *Cecidomyiidae* 2 | *Cecidomyiidae sp.* 2 | 1860 |  |
| Diptera | Cecidomyiidae | *Cecidomyiidae* 3 | *Cecidomyiidae sp.* 3 | 1860 |  |
| Diptera | Ceratopogonidae | *Ceratopogonidae* | *Ceratopogonidae sp.* | 1181, 1463, 1632, 1860, 2283 |  |
| Diptera | Chaoboridae | *Chaoboridae* | *Chaoboridae sp.* | 1463 |  |
| Diptera | Chironomidae | *Chironomidae* | *Chironomidae sp.* | 1181, 1463, 1860, 2080, 2283, 2379, 2587 |  |
| Diptera | Chloropidae | *Chloropidae* | *Chloropidae sp.* | 1181, 1463, 1632, 1860, 2080, 2283, 2379, 2587 |  |
| Diptera | Dolichopodidae | *Dolichopodidae* | *Dolichopodidae sp.* | 1181, 1463, 1632, 2080, 2283 |  |
| Diptera | Empididae | *Empididae* | *Empididae sp.* | 1181, 1463, 1632, 1860, 2080, 2283, 2379, 2587 | X |
| Diptera | Ephydridae | *Ephydridae* | *Ephydridae sp.* | 1181, 1463, 1632, 1860, 2080, 2283, 2379 |  |
| Diptera | Fanniidae | *Fannia* | *Fannia sp.* | 2080 | X |
| Diptera | Fanniidae | *Fanniidae* | *Fanniidae sp.* | 1463, 1860, 2080, 2587 | X |
| Diptera | Hybotidae | *Hybotidae* | *Hybotidae sp.* | 1632, 1860, 2283, 2587 |  |
| Diptera | Lauxaniidae | *Lauxaniidae* | *Lauxaniidae sp.* | 1463, 2283 |  |
| Diptera | Lonchaeidae | *Lonchaeidae* | *Lonchaeidae sp.* | 1463 |  |
| Diptera | Lonchopteridae | *Lonchopteridae* | *Lonchopteridae sp.* | 1463, 1632, 1860 |  |
| Diptera | Milichiidae | *Milichiidae* | *Milichiidae sp.* | 1181, 2379 |  |
| Diptera | Muscidae | *Coenosia* | *Coenosia alpicola* | 1181, 1463 | X |
| Diptera | Muscidae | *Coenosia* | *Coenosia ambigua* | 1181, 1860 |  |
| Diptera | Muscidae | *Coenosia* | *Coenosia bilineella* | 1181 | X |
| Diptera | Muscidae | *Coenosia* | *Coenosia intermedia* | 1181 | X |
| Diptera | Muscidae | *Coenosia* | *Coenosia lineatipes* | 1181 |  |
| Diptera | Muscidae | *Coenosia* | *Coenosia obscuricula* | 1860, 2283 | X |
| Diptera | Muscidae | *Coenosia* | *Coenosia sp.* | 1181, 1463, 1632, 2080, 2283, 2379, 2587 |  |
| Diptera | Muscidae | *Drymeia* | *Drymeia alpicola* | 1632, 2080, 2283 |  |
| Diptera | Muscidae | *Drymeia* | *Drymeia brumalis* | 1632 |  |
| Diptera | Muscidae | *Drymeia* | *Drymeia hamata* | 1181, 1860 | X |
| Diptera | Muscidae | *Drymeia* | *Drymeia sp.* | 1181, 1463, 1860, 2080, 2283, 2379 | X |
| Diptera | Muscidae | *Haematobia* | *Haematobia irritans* | 1181, 1632 |  |
| Diptera | Muscidae | *Haematobia* | *Haematobia titillans* | 1181 |  |
| Diptera | Muscidae | *Helina* | *Helina reversio* | 2283, 2379 | X |
| Diptera | Muscidae | *Hydrotaea* | *Hydrotaea albipuncta* | 1463 | X |
| Diptera | Muscidae | *Morellia* | *Morellia podagrica* | 1181 | X |
| Diptera | Muscidae | *Musca* | *Musca autumnalis* | 1181, 1463, 1860, 2283 |  |
| Diptera | Muscidae | *Muscidae* | *Muscidae sp.* | 1181, 1463, 1632, 1860 |  |
| Diptera | Muscidae | *Phaonia* | *Phaonia angelicae* | 1181 |  |
| Diptera | Muscidae | *Phaonia* | *Phaonia lugubris* | 1860 | X |
| Diptera | Muscidae | *Phaonia* | *Phaonia serva* | 1463, 1632 | X |
| Diptera | Muscidae | *Phaonia* | *Phaonia sp.* | 1860 |  |
| Diptera | Muscidae | *Thricops* | *Thricops aculeipes* | 1860, 2283 | X |
| Diptera | Muscidae | *Thricops* | *Thricops culminum* | 1860, 2283 | X |
| Diptera | Muscidae | *Thricops* | *Thricops cunctans* | 1463 | X |
| Diptera | Muscidae | *Thricops* | *Thricops furcatus* | 1632 | X |
| Diptera | Muscidae | *Thricops* | *Thricops longipes* | 1181, 1860, 2283 |  |
| Diptera | Muscidae | *Thricops* | *Thricops nigritellus* | 1860 | X |
| Diptera | Muscidae | *Thricops* | *Thricops sp.* | 1181, 1463, 1632, 1860, 2080, 2283, 2379, 2587 | X |
| Diptera | Muscidae | *Muscidae* 10 | *Muscidae sp.* 10 | 1181, 1632, 1860, 2080, 2283, 2379, 2587 | X |
| Diptera | Muscidae | *Muscidae* 11 | *Muscidae sp.* 11 | 1181, 1463, 1632, 1860, 2080, 2283, 2379, 2587 |  |
| Diptera | Muscidae | *Muscidae* 2 | *Muscidae sp.* 2 | 1860 |  |
| Diptera | Muscidae | *Muscidae* 3 | *Muscidae sp.* 3 | 1181, 1632, 1860, 2080, 2283 | X |
| Diptera | Muscidae | *Muscidae* 4 | *Muscidae sp.* 4 | 1463, 1860, 2080 | X |
| Diptera | Muscidae | *Muscidae* 5 | *Muscidae sp.* 5 | 1463, 1632, 1860, 2080, 2283, 2379, 2587 | X |
| Diptera | Muscidae | *Muscidae* 8 | *Muscidae sp.* 8 | 1181 | X |
| Diptera | Muscidae | *Muscidae* 9 | *Muscidae sp.* 9 | 1181, 1463, 1632, 1860, 2080, 2283, 2379, 2587 | X |
| Diptera | Mycetophilidae | *Mycetophilidae* | *Mycetophilidae sp.* | 1181 |  |
| Diptera | Opomyzidae | *Geomyza* | *Geomyza sp.* | 1632 |  |
| Diptera | Phoridae | *Conicera* | *Conicera floricola* | 1463 |  |
| Diptera | Phoridae | *Conicera* | *Conicera sp.* | 1463 |  |
| Diptera | Phoridae | *Diplonevra* | *Diplonevra crassicornis* | 2283 |  |
| Diptera | Phoridae | *Diplonevra* | *Diplonevra glabra* | 1181 |  |
| Diptera | Phoridae | *Megaselia* | *Megaselia abdita* | 2283 |  |
| Diptera | Phoridae | *Megaselia* | *Megaselia aculeata* | 1463 |  |
| Diptera | Phoridae | *Megaselia* | *Megaselia brevicostalis* | 1632, 2379 |  |
| Diptera | Phoridae | *Megaselia* | *Megaselia ciliata* | 1463 |  |
| Diptera | Phoridae | *Megaselia* | *Megaselia nectergata* | 1463, 1860, 2080 |  |
| Diptera | Phoridae | *Megaselia* | *Megaselia pectoralis* | 2080, 2283, 2379 |  |
| Diptera | Phoridae | *Megaselia* | *Megaselia pusilla* | 2379 |  |
| Diptera | Phoridae | *Megaselia* | *Megaselia sordida* | 2283, 2379 |  |
| Diptera | Phoridae | *Megaselia* | *Megaselia sp.* | 1181, 1463, 1632, 1860, 2080, 2283, 2379, 2587 |  |
| Diptera | Phoridae | *Megaselia* | *Megaselia subfuscipes* | 1463, 2283 |  |
| Diptera | Phoridae | *Megaselia* | *Megaselia subnudipennis* | 2283 |  |
| Diptera | Phoridae | *Phora* | *Phora sp.* | 1463 | X |
| Diptera | Phoridae | *Phora* | *Phora stictica* | 1181, 1463, 2080 |  |
| Diptera | Phoridae | *Phoridae* | *Phoridae sp.* | 1181, 1463, 1632, 1860, 2283, 2379, 2587 |  |
| Diptera | Phoridae | *Triphleba* | *Triphleba inaequalis* | 2379 |  |
| Diptera | Phoridae | *Triphleba* | *Triphleba luteifemorata* | 1463 |  |
| Diptera | Phoridae | *Triphleba* | *Triphleba sp.* | 1181, 1632 |  |
| Diptera | Piophilidae | *Piophilidae* | *Piophilidae sp.* | 1860 | X |
| Diptera | Pipunculidae | *Pipunculidae* | *Pipunculidae sp.* | 1463 |  |
| Diptera | Platypezidae | *Platypezidae* | *Platypezidae sp.* | 1860 |  |
| Diptera | Psilidae | *Psilidae* | *Psilidae sp.* | 1632, 2283, 2379 |  |
| Diptera | Rhagionidae | *Rhagionidae* | *Rhagionidae sp.* | 1181, 1463 | X |
| Diptera | Rhinophoridae | *Rhinophoridae* | *Rhinophoridae sp.* | 1181 | X |
| Diptera | Sarcophagidae | *Sarcophaga* | *Sarcophaga haemorrhoa* | 1181 | X |
| Diptera | Sarcophagidae | *Sarcophaga* | *Sarcophaga vagans* | 1632 | X |
| Diptera | Sarcophagidae | *Sarcophagidae* | *Sarcophagidae sp.* | 1181, 1463, 1632, 1860, 2080, 2283 | X |
| Diptera | Scathophagidae | *Scathophaga* | *Scathophaga stercoraria* | 2283 |  |
| Diptera | Scathophagidae | *Scathophagidae* | *Scathophagidae sp.* | 1181, 1463, 1632, 1860, 2080, 2283, 2379, 2587 | X |
| Diptera | Scatopsidae | *Scatopsidae* | *Scatopsidae sp.* | 1181, 1463, 2283 |  |
| Diptera | Sciaridae | *Bradysia* | *Bradysia alpicola* | 1463 |  |
| Diptera | Sciaridae | *Bradysia* | *Bradysia flavipila* | 1860 |  |
| Diptera | Sciaridae | *Bradysia* | *Bradysia pauperata* | 2587 | X |
| Diptera | Sciaridae | *Bradysia* | *Bradysia polonica* | 1463 |  |
| Diptera | Sciaridae | *Bradysia* | *Bradysia praecox* | 1181, 1860, 2379 |  |
| Diptera | Sciaridae | *Bradysia* | *Bradysia sp.* | 1181, 1463, 1632, 1860, 2080, 2283, 2379, 2587 |  |
| Diptera | Sciaridae | *Bradysia* | *Bradysia subscabricornis* | 2283 |  |
| Diptera | Sciaridae | *Bradysia* | *Bradysia zetterstedti* | 1860 |  |
| Diptera | Sciaridae | *Camptochaeta* | *Camptochaeta austriaca* | 2283 |  |
| Diptera | Sciaridae | *Claustropyga* | *Claustropyga refrigerata* | 1860 |  |
| Diptera | Sciaridae | *Corynoptera* | *Corynoptera sp.* | 1463, 1860, 2080, 2283 |  |
| Diptera | Sciaridae | *Cratyna* | *Cratyna colei* | 1181 |  |
| Diptera | Sciaridae | *Cratyna* | *Cratyna nobilis* | 1463 |  |
| Diptera | Sciaridae | *Cratyna* | *Cratyna sp.* | 1632, 1860, 2283, 2379 |  |
| Diptera | Sciaridae | *Epidapus* | *Epidapus microthorax* | 1181 |  |
| Diptera | Sciaridae | *Lycoriella* | *Lycoriella inflata* | 2283 | X |
| Diptera | Sciaridae | *Peyerimhoffia* | *Peyerimhoffia alpina* | 2283 |  |
| Diptera | Sciaridae | *Sciara* | *Sciara flavimana* | 1181 |  |
| Diptera | Sciaridae | *Sciara* | *Sciara hemerobioides* | 1463 | X |
| Diptera | Sciaridae | *Sciara* | *Sciara sp.* | 1463 | X |
| Diptera | Sciaridae | *Sciaridae* | *Sciaridae sp.* | 1181, 1463, 1860, 2080, 2283, 2379 |  |
| Diptera | Sepsidae | *Orygma* | *Orygma luctuosum* | 2080 |  |
| Diptera | Sepsidae | *Saltella* | *Saltella sphondylii* | 1181, 1463, 1632, 1860, 2080 |  |
| Diptera | Sepsidae | *Sepsidae* | *Sepsidae sp.* | 1860, 2283 |  |
| Diptera | Sepsidae | *Sepsis* | *Sepsis cynipsea* | 1181, 1463, 1632, 1860 |  |
| Diptera | Sepsidae | *Sepsis* | *Sepsis flavimana* | 1463 |  |
| Diptera | Sepsidae | *Sepsis* | *Sepsis neocynipsea* | 1181, 1463, 1632, 1860, 2283, 2379, 2587 |  |
| Diptera | Sepsidae | *Themira* | *Themira annulipes* | 1181, 1632 |  |
| Diptera | Sepsidae | *Themira* | *Themira lucida* | 2080 |  |
| Diptera | Sepsidae | *Themira* | *Themira minor* | 1463, 1632, 1860, 2283 |  |
| Diptera | Sepsidae | *Themira* | *Themira superba* | 1181, 1463, 2283 |  |
| Diptera | Sepsidae | *Themira* | *Themira leachi* | 1463, 1860, 2283, 2379 | X |
| Diptera | Sphaeroceridae | *Sphaeroceridae* | *Sphaeroceridae sp.* | 1181 |  |
| Diptera | Stratiomyidae | *Stratiomyidae* | *Stratiomyidae sp.* | 1181, 1860 | X |
| Diptera | Syrphidae | *Arctophila* | *Arctophila bombiforme* | 1181 | X |
| Diptera | Syrphidae | *Cheilosia* | *Cheilosia carbonaria* | 1181 | X |
| Diptera | Syrphidae | *Cheilosia* | *Cheilosia griseiventris* | 1632 | X |
| Diptera | Syrphidae | *Cheilosia* | *Cheilosia laticornis* | 1632 | X |
| Diptera | Syrphidae | *Cheilosia* | *Cheilosia sp.* | 1181, 1463, 1632, 2283, 2379 | X |
| Diptera | Syrphidae | *Chrysotoxum* | *Chrysotoxum fasciolatum* | 1181 | X |
| Diptera | Syrphidae | *Chrysotoxum* | *Chrysotoxum sp.* | 1181 | X |
| Diptera | Syrphidae | *Epistrophe* | *Epistrophe sp.* | 1181, 1632, 2080, 2283 |  |
| Diptera | Syrphidae | *Episyrphus* | *Episyrphus balteatus* | 1181, 1463, 1632, 1860, 2080, 2283, 2587 | X |
| Diptera | Syrphidae | *Eristalis* | *Eristalis horticola* | 2283 | X |
| Diptera | Syrphidae | *Eristalis* | *Eristalis jugorum* | 1463 | X |
| Diptera | Syrphidae | *Eristalis* | *Eristalis rupium* | 1181 | X |
| Diptera | Syrphidae | *Eristalis* | *Eristalis sp.* | 1181, 1463, 1632 | X |
| Diptera | Syrphidae | *Eristalis* | *Eristalis tenax* | 1181, 1463, 1632, 2283 | X |
| Diptera | Syrphidae | *Eupeodes* | *Eupeodes sp.* | 1181, 1632, 2080, 2283, 2379, 2587 | X |
| Diptera | Syrphidae | *Melanostoma* | *Melanostoma sp.* | 1632, 2283 |  |
| Diptera | Syrphidae | *Myolepta* | *Myolepta obscura* | 1181 | X |
| Diptera | Syrphidae | *Platycheirus* | *Platycheirus sp.* | 1181, 1632, 2283, 2379 | X |
| Diptera | Syrphidae | *Rhingia* | *Rhingia campestris* | 1860, 2283 | X |
| Diptera | Syrphidae | *Rhingia* | *Rhingia sp.* | 1181, 1632 | X |
| Diptera | Syrphidae | *Scaeva* | *Scaeva sp.* | 1181, 1632, 1860, 2283, 2379 |  |
| Diptera | Syrphidae | *Sphaerophoria* | *Sphaerophoria scripta* | 1181, 1632 | X |
| Diptera | Syrphidae | *Sphaerophoria* | *Sphaerophoria shirchan* | 1632 | X |
| Diptera | Syrphidae | *Syrphidae* | *Syrphidae sp.* | 1181, 1463, 1632, 1860, 2080, 2283, 2379, 2587 | X |
| Diptera | Syrphidae | *Syrphus* | *Syrphus admirandus* | 1181, 1463, 2283 | X |
| Diptera | Syrphidae | *Syrphus* | *Syrphus auberti* | 1181 | X |
| Diptera | Syrphidae | *Syrphus* | *Syrphus sp.* | 1181, 1632, 2283 | X |
| Diptera | Syrphidae | *Syrphus* | *Syrphus torvus* | 2283 | X |
| Diptera | Syrphidae | *Syrphus* | *Syrphus vitripennis* | 1181 |  |
| Diptera | Syrphidae | *Dasysyrphus* | *Dasysyrphus sp.* | 2283 | X |
| Diptera | Tachinidae | *Dinera* | *Dinera carinifrons* | 1632 | X |
| Diptera | Tachinidae | *Estheria* | *Estheria bohemani* | 1463 | X |
| Diptera | Tachinidae | *Macquartia* | *Macquartia tenebricosa* | 1860 |  |
| Diptera | Tachinidae | *Meigenia* | *Meigenia mutabilis* | 1181 | X |
| Diptera | Tachinidae | *Siphona* | *Siphona sp.* | 1181, 1632 | X |
| Diptera | Tachinidae | *Tachinidae* | *Tachinidae sp.* | 1181, 1632 | X |
| Diptera | Therevidae | *Therevidae* | *Therevidae sp.* | 2283, 2587 | X |
| Diptera | Tipulidae | *Tipulidae* | *Tipulidae sp.* | 1463, 1632, 1860, 2283, 2379 | X |
| Diptera | Ulidiidae | *Ulidiidae* | *Ulidiidae sp.* | 1860 |  |
| Diptera | Diptera | *Diptera* | *Diptera sp.* | 1860 | X |
| Diptera | Odiniidae | *Odinidae* | *Odinidae sp.* | 2283 |  |
| Hemiptera | Aphididae | *Aphididae* | *Aphididae sp.* | 1181, 1463, 1860, 2080 |  |
| Hemiptera | Auchenorrhyncha | *Auchenorrhyncha* | *Auchenorrhyncha sp.* | 1181, 1632 |  |
| Hemiptera | Delphacidae | *Delphacidae* | *Delphacidae sp.* | 1181, 1860 |  |
| Hemiptera | Issidae | *Issidae* | *Issidae sp.* | 1181, 1463, 1632, 1860, 2080, 2283, 2379 |  |
| Hemiptera | Lygaeidae | *Lygaeidae* | *Lygaeidae sp.* | 1181, 1463 |  |
| Hemiptera | Miridae | *Miridae* | *Miridae sp.* | 1181, 1463, 1632, 1860 | X |
| Hemiptera | Nabidae | *Nabidae* | *Nabidae sp.* | 1463 | X |
| Hemiptera | Saldidae | *Saldidae* | *Saldidae sp.* | 1181, 2379 |  |
| Hemiptera | Aradidae | *Aradidae* | *Aradidae sp.* | 1181 |  |
| Hymenoptera | Apidae | *Apidae* | *Apidae sp.* | 1181, 1463, 1632, 1860, 2587 | X |
| Hymenoptera | Apidae | *Apis* | *Apis mellifera* | 1181, 1463 | X |
| Hymenoptera | Apidae | *Bombus* | *Bombus gerstaeckeri* | 1181, 1632 | X |
| Hymenoptera | Apidae | *Bombus* | *Bombus hortorum group* | 1181, 2283 | X |
| Hymenoptera | Apidae | *Bombus* | *Bombus humilis* | 1181 |  |
| Hymenoptera | Apidae | *Bombus* | *Bombus jonellus* | 1181, 1632, 2283 | X |
| Hymenoptera | Apidae | *Bombus* | *Bombus pascuorum* | 1181, 1463, 1632 | X |
| Hymenoptera | Apidae | *Bombus* | *Bombus quadricolor* | 1463 | X |
| Hymenoptera | Apidae | *Bombus* | *Bombus ruderarius* | 1181, 1463, 1632, 2080, 2283 | X |
| Hymenoptera | Apidae | *Bombus* | *Bombus rupestris* | 1463 | X |
| Hymenoptera | Apidae | *Bombus* | *Bombus sichelii* or *pyrenaeus* | 1181, 1463, 1632, 1860, 2080, 2283 | X |
| Hymenoptera | Apidae | *Bombus* | *Bombus soroeensis* | 1181, 1632, 1860, 2080, 2283 | X |
| Hymenoptera | Apidae | *Bombus* | *Bombus sp.* | 1181, 2283 | X |
| Hymenoptera | Apidae | *Bombus* | *Bombus subterraneus* | 1463 | X |
| Hymenoptera | Apidae | *Bombus* | *Bombus terrestris group* | 1181, 1463, 1632, 2283 | X |
| Hymenoptera | Apidae | *Bombus* | *Bombus wurflenii* | 1181, 2283 | X |
| Hymenoptera | Apidae | *Nomada* | *Nomada flavoguttata* | 1181 | X |
| Hymenoptera | Braconidae | *Braconidae* | *Braconidae sp.* | 1181, 1463, 1632, 1860, 2283, 2379, 2587 |  |
| Hymenoptera | Chalcidoidea | *Chalcidoidea* | *Chalcidoidea sp.* | 1181, 1463, 1860, 2283, 2379 |  |
| Hymenoptera | Cimbicidae | *Cimbicidae* | *Cimbicidae sp.* | 2283 | X |
| Hymenoptera | Crabronidae | *Mellinus* | *Mellinus arvensis* | 1463 | X |
| Hymenoptera | Diapriidae | *Diapriidae* | *Diapriidae sp.* | 2283, 2587 |  |
| Hymenoptera | Formicidae | *Formica* | *Formica exsecta* | 1181, 1463 |  |
| Hymenoptera | Formicidae | *Formica* | *Formica lemani* | 1181 |  |
| Hymenoptera | Formicidae | *Formica* | *Formica polyctena* | 1463 |  |
| Hymenoptera | Formicidae | *Formica* | *Formica rufa* | 1181 |  |
| Hymenoptera | Formicidae | *Formica* | *Formica transkaukasica* | 1181, 1463, 1632, 1860 |  |
| Hymenoptera | Formicidae | *Formicidae* | *Formicidae sp.* | 1463 |  |
| Hymenoptera | Formicidae | *Lasius* | *Lasius sp.* | 1181 | X |
| Hymenoptera | Formicidae | *Leptothorax* | *Leptothorax sp.* | 1181, 1463, 1860, 2080 |  |
| Hymenoptera | Ichneumonidae | *Ichneumonidae* | *Ichneumonidae sp.* | 1181, 1463, 1632, 1860, 2080, 2283, 2379, 2587 |  |
| Hymenoptera | Megaspilidae | *Megaspilidae* | *Megaspilidae sp.* | 1463, 2283 |  |
| Hymenoptera | Pamphiliidae | *Pamphiliidae* | *Pamphiliidae sp.* | 1463, 1860 | X |
| Hymenoptera | Platygastridae | *Platygastridae* | *Platygastridae sp.* | 1181, 2379 |  |
| Hymenoptera | Platygastridae | *Scelionidae* | *Scelionidae sp.* | 1463 |  |
| Hymenoptera | Pompilidae | *Pompilidae* | *Pompilidae sp.* | 1181, 1463 |  |
| Hymenoptera | Proctotrupidae | *Proctotrupidae* | *Proctotrupidae sp.* | 1860 |  |
| Hymenoptera | Tenthredinidae | *Tenthredinidae* | *Tenthredinidae sp.* | 1181, 1463, 1632, 1860, 2080, 2283, 2379 | X |
| Hymenoptera | Xiphydriidae | *Xiphydriidae* | *Xiphydriidae sp.* | 2283 |  |
| Hymenoptera | Xyelidae | *Xyelidae* | *Xyelidae sp.* | 2080 | X |
| Hymenoptera | Xylophagidae | *Xylophagidae* | *Xylophagidae sp.* | 1860 |  |
| Hymenoptera | Figitidae | *Figitidae* | *Figitidae sp.* | 1181, 1463, 1632, 2283 |  |
| Hymenoptera | Andrenidae | *Andrena* | *Andrena bicolor* | 1463 | X |
| Hymenoptera | Andrenidae | *Andrena* | *Andrena cineraria* | 1181 | X |
| Hymenoptera | Andrenidae | *Andrena* | *Andrena coitana* | 1181, 1632 |  |
| Hymenoptera | Andrenidae | *Andrena* | *Andrena hattorfiana* | 1181 | X |
| Hymenoptera | Andrenidae | *Andrena* | *Andrena minutula* | 1181, 1463 | X |
| Hymenoptera | Andrenidae | *Andrena* | *Andrena rogenhoferi* | 2379 | X |
| Hymenoptera | Andrenidae | *Andrena* | *Andrena subopaca* | 1181, 1463 | X |
| Hymenoptera | Andrenidae | *Andrena* | *Andrena carantonica* | 2379 | X |
| Hymenoptera | Halictidae | *Halictus* | *Halictus rubicundus* | 1181, 1463, 1632, 2080 | X |
| Hymenoptera | Halictidae | *Lasioglossum* | *Lasioglossum alpibes* | 1181, 1632 | X |
| Hymenoptera | Halictidae | *Lasioglossum* | *Lasioglossum calceatum* | 1181, 1632 | X |
| Hymenoptera | Halictidae | *Lasioglossum* | *Lasioglossum fratellum* | 1181, 1632 | X |
| Hymenoptera | Halictidae | *Lasioglossum* | *Lasioglossum fulvicorne* | 1181, 1463 | X |
| Hymenoptera | Halictidae | *Lasioglossum* | *Lasioglossum laevigatum* | 1632 | X |
| Hymenoptera | Halictidae | *Lasioglossum* | *Lasioglossum leucopus* | 1181, 1463 | X |
| Hymenoptera | Halictidae | *Lasioglossum* | *Lasioglossum sp.* | 1463 | X |
| Hymenoptera | Megachilidae | *Anthidium* | *Anthidium byssinum* | 1181 | X |
| Hymenoptera | Colletidae | *Hylaeus* | *Hylaeus communis* | 1632 | X |
| Hymenoptera | Colletidae | *Hylaeus* | *Hylaeus confusus* | 1181 | X |
| Lepidoptera | Adelidae | *Cauchas* | *Cauchas fibulella* | 1463 |  |
| Lepidoptera | Coleophoridae | *Coleophora* | *Coleophora sp.* | 1181 |  |
| Lepidoptera | Crambidae | *Agriphila* | *Agriphila sp.* | 1181, 1463 |  |
| Lepidoptera | Crambidae | *Agriphila* | *Agriphila straminella* | 1463 |  |
| Lepidoptera | Crambidae | *Catoptria* | *Catoptria petrificella* or *combinella* | 1860, 2080 |  |
| Lepidoptera | Crambidae | *Chrysoteuchia* | *Chrysoteuchia culmella* | 1181 |  |
| Lepidoptera | Crambidae | *Crambinae* | *Crambinae sp.* | 1181 |  |
| Lepidoptera | Crambidae | *Crambus* | *Crambus cf lathoniellus* | 1181 |  |
| Lepidoptera | Crambidae | *Eudonia* | *Eudonia vallesialis* | 2587 |  |
| Lepidoptera | Crambidae | *Udea* | *Udea alpinalis* | 1632 |  |
| Lepidoptera | Crambidae | *Udea* | *Udea uliginosalis* | 2283 |  |
| Lepidoptera | Gelechiidae | *Gelechiidae* 1 | *Gelechiidae sp.* 1 | 1463 |  |
| Lepidoptera | Gelechiidae | *Gelechiidae* 2 | *Gelechiidae sp.* 2 | 1463 |  |
| Lepidoptera | Gelechiidae | *Gelechiidae* 3 | *Gelechiidae sp.* 3 | 1181 |  |
| Lepidoptera | Geometridae | *Geometridae* | *Geometridae or Crambidae* | 2283 |  |
| Lepidoptera | Geometridae | *Glacies* | *Glacies alpinata* | 1860 |  |
| Lepidoptera | Geometridae | *Glacies* | *Glacies coracina* | 2379 |  |
| Lepidoptera | Geometridae | *Glacies* | *Glacies noricana* | 2379 |  |
| Lepidoptera | Geometridae | *Glacies* | *Glacies sp.* | 2379 |  |
| Lepidoptera | Geometridae | *Macaria* | *Macaria brunneata* | 1632 |  |
| Lepidoptera | Geometridae | *Mesotype* | *Mesotype verberata* | 2283 |  |
| Lepidoptera | Geometridae | *Perizoma* | *Perizoma albulata* | 2283 |  |
| Lepidoptera | Geometridae | *Xanthorhoe* | *Xanthorhoe montanata* | 1860 |  |
| Lepidoptera | Glyphipterigidae | *Glyphipterix* | *Glyphipterix simpliciella* | 1463 |  |
| Lepidoptera | Hesperiidae | *Hesperia* | *Hesperia comma* | 1181 |  |
| Lepidoptera | Lepidoptera | *Lepidoptera* | *Lepidoptera sp.* | 1463, 2080, 2379 |  |
| Lepidoptera | Lycaenidae | *Plebejus* | *Plebejus argus* | 1181 |  |
| Lepidoptera | Lycaenidae | *Vacciniina* | *Vacciniina optilete* | 2080 |  |
| Lepidoptera | Micropterigidae | *Micropterix* | *Micropterix aruncella* | 1181, 1632 |  |
| Lepidoptera | Nymphalidae | *Boloria* | *Boloria pales* | 2283 | X |
| Lepidoptera | Nymphalidae | *Erebia* | *Erebia epiphron* | 1632, 2283 |  |
| Lepidoptera | Nymphalidae | *Erebia* | *Erebia manto* | 2283 | X |
| Lepidoptera | Nymphalidae | *Erebia* | *Erebia manto* or *eriphyle* | 2283 | X |
| Lepidoptera | Nymphalidae | *Erebia* | *Erebia sp.* | 2283 | X |
| Lepidoptera | Nymphalidae | *Melitaea* | *Melitaea asteria* | 2379 | X |
| Lepidoptera | Pterophoridae | *Pterophoridae* | *Pterophoridae sp.* | 1463 |  |
| Lepidoptera | Pyralidae | *Asarta* | *Asarta aethiopella* | 2283 |  |
| Lepidoptera | Pyralidae | *Pempeliella* | *Pempeliella ornatella* | 1181 |  |
| Lepidoptera | Scythrididae | *Scythris* | *Scythris sp.* | 2283, 2379, 2587 |  |
| Lepidoptera | Tortricidae | *Ancylis* | *Ancylis unguicella* | 2379 |  |
| Lepidoptera | Tortricidae | *Aterpia* | *Aterpia corticana* | 1860 |  |
| Lepidoptera | Tortricidae | *Eana* | *Eana osseana* | 1860, 2283 |  |
| Lepidoptera | Tortricidae | *Epinotia* | *Epinotia mercuriana* | 2080, 2283, 2379 |  |
| Lepidoptera | Tortricidae | *Phiaris* | *Phiaris micana* | 1632 |  |
| Lepidoptera | Tortricidae | *Tortricidae* | *Tortricidae sp.* | 1860, 2587 |  |
| Lepidoptera | Zygaenidae | *Zygaena* | *Zygaena exulans* | 2283, 2379 | X |
| Lepidoptera | Zygaenidae | *Zygaena* | *Zygaena purpuralis* | 1181 |  |
| Mecoptera | Bittacidae | *Bittacidae* | *Bittacidae sp.* | 1463 |  |
| Mecoptera | Panorpidae | *Panorpa* | *Panorpa sp.* | 1632 |  |
| Neuroptera | Chrysopidae | *Chrysoperla* | *Chrysoperla sp.* | 1463 |  |
| Orthoptera | Acrididae | *Chorthippus* | *Chorthippus sp.* | 2080 |  |
| Orthoptera | Acrididae | *Chorthippus* | *Chorthippus montanus* | 1860 |  |
| Psocoptera | Lachesillidae | *Lachesillidae* | *Lachesillidae sp.* | 1463, 2080 |  |
| Psocoptera | Peripsocidae | *Peripsocidae* | *Peripsocidae sp.* | 1181, 1463, 1860, 2283 |  |
| Raphidioptera | Raphidiidae | *Raphidiidae* | *Raphidiidae sp.* | 1463 | X |
| Thysanoptera | Aeolothripidae | *Aeolothripidae* | *Aeolothripidae sp.* | 2080 |  |
| Thysanoptera | Thripidae | *Thripidae* | *Thripidae sp.* | 1181, 2080, 2283 |  |

**Table S2** List of flowering plants collected in all 24 communities and elevation at which they are found

| **Taxonomic Level** | **Elevation** |
| --- | --- |
| **Species** |  |
| *Achillea atrata* | 2283 |
| *Achillea millefolium* | 1181, 1463, 1632, 1860 |
| *Aconitum tauricum* | 2283 |
| *Adenostyles alliariae* | 2283 |
| *Ajuga pyramidalis* | 1632 |
| *Ajuga reptans* | 1181, 1463 |
| *Alchemilla coriacea* | 2080 |
| *Alchemilla xanthochlora* | 1181, 1463, 1632, 1860, 2080, 2283 |
| *Androsace obtusifolia* | 2283, 2379 |
| *Anemone alpina* | 1632, 1860, 2080, 2283 |
| *Antennaria carpatica* | 2283 |
| *Arabis alpina* | 2283, 2587 |
| *Arabis soyeri* | 2283 |
| *Arenaria ciliata* | 2587 |
| *Arnica montana* | 2283 |
| *Aster bellidiastrum* | 1632, 1860, 2283, 2379 |
| *Astragalus alpinus* | 2587 |
| *Astragalus norvegicus* | 2283 |
| *Bartsia alpina* | 2283, 2379 |
| *Bellis perennis* | 1181, 1463 |
| *Biscutella laevigata* | 1181 |
| *Calluna vulgaris* | 1181, 1463, 1860, 2080 |
| *Caltha palustris* | 2080 |
| *Campanula barbata* | 2080, 2283 |
| *Campanula rotundifolia* | 1181, 1463, 1632, 1860 |
| *Campanula scheuchzeri* | 2080, 2283, 2379 |
| *Capsella bursa pastoris* | 1463 |
| *Cardamine amara* | 1632, 1860 |
| *Carduus defloratus* | 1463 |
| *Carlina acaulis* | 1463 |
| *Carum carvi* | 1181, 1463, 1860 |
| *Centaurea jacea* | 1181 |
| *Cerastium fontanum* | 1181, 1860, 2283, 2379 |
| *Cerastium uniflorum* | 2379, 2587 |
| *Chrysosplenium alternifolium* | 1632 |
| *Cirsium acaule* | 1181 |
| *Cirsium spinosissimum* | 2283 |
| *Clinopodium acinos* | 1181, 1463 |
| *Comastoma nanum* | 2587 |
| *Crepis aurea* | 1181, 1463, 1860, 2080, 2283, 2379 |
| *Crepis conyzifolia* | 1463 |
| *Dactylorhiza maculata* | 1181 |
| *Dianthus glacialis* | 2283, 2379 |
| *Doronicum glaciale* | 2283, 2379, 2587 |
| *Dryas octopetala* | 2379 |
| *Epilobium alsinifolium* | 2283 |
| *Erigeron uniflorus* | 2283, 2587 |
| *Euphrasia minima* | 1181, 1463, 1860, 2080, 2283, 2379, 2587 |
| *Euphrasia officinalis* | 1181, 1632 |
| *Euphrasia rostkoviana* | 2080 |
| *Ficaria verna* | 1632 |
| *Fragaria vesca* | 1181, 1463 |
| *Gagea serotina* | 2283, 2379 |
| *Galeopsis tetrahit* | 1463 |
| *Galium anisophyllon* | 2283, 2379 |
| *Galium mollugo* | 1181, 1463, 1632 |
| *Galium pumilum* | 1463, 1632, 1860 |
| *Gentiana acaulis* | 1860, 2080, 2283 |
| *Gentiana nivalis* | 2283 |
| *Gentiana verna* | 1632, 1860, 2080, 2283, 2379, 2587 |
| *Gentianella germanica* | 2283 |
| *Geranium sylvaticum* | 1463, 1860 |
| *Geum montanum* | 1860, 2080, 2283 |
| *Gymnadenia nigra* | 2283 |
| *Helianthemum oelandicum* | 2379 |
| *Hieracium alpinum* | 1860, 2080 |
| *Hieracium hoppeanum* | 1181 |
| *Hieracium murorum* | 1632 |
| *Homogyne alpina* | 1181, 1463, 1632, 1860, 2080, 2379 |
| *Hypericum maculatum* | 1181, 1463, 1632, 2080 |
| *Leontodon hispidus* | 1181, 1463, 1632, 1860, 2080, 2283, 2379 |
| *Leucanthemopsis alpina* | 2587 |
| *Leucanthemum vulgare* | 1181, 1463, 1632 |
| *Ligusticum mutellina* | 2283, 2379 |
| *Linaria alpina* | 2283, 2587 |
| *Loiseleuria procumbens* | 1860, 2080, 2379 |
| *Lotus corniculatus* | 1181, 1463 |
| *Maianthemum bifolium* | 1463 |
| *Minuartia rupestris* | 2587 |
| *Minuartia sedoides* | 2379, 2587 |
| *Minuartia verna* | 2379 |
| *Moehringia ciliata* | 2587 |
| *Myosotis alpestris* | 2283, 2379, 2587 |
| *Myosotis sylvatica* | 1463, 1632 |
| *Parnassia palustris* | 1181, 1463, 2283, 2379 |
| *Pedicularis asplenifolia* | 2379, 2587 |
| *Persicaria vivipara* | 1860, 2080, 2283, 2379, 2587 |
| *Phyteuma globulariifolium* | 2587 |
| *Phyteuma hemisphaericum* | 1632, 1860, 2080 |
| *Phyteuma orbiculare* | 2283, 2379 |
| *Pilosella lactucella* | 1463 |
| *Pilosella officinarum* | 1463 |
| *Pimpinella major* | 1181, 1463 |
| *Plantago lanceolata* | 1181, 1463 |
| *Plantago media* | 1181, 1463, 1632 |
| *Polygala vulgaris* | 1181, 1463, 1632 |
| *Potentilla aurea* | 1181, 1463, 1632, 1860, 2080, 2283 |
| *Potentilla crantzii* | 2283 |
| *Potentilla erecta* | 1181, 1463, 1632, 1860 |
| *Potentilla reptans* | 1181 |
| *Primula minima* | 1860, 2080, 2283 |
| *Pritzelago alpina* | 2587 |
| *Prunella vulgaris* | 1181, 1463, 1632 |
| *Pseudorchis albida* | 1860 |
| *Ranunculus acris* | 1181, 1463, 1632, 1860, 2080 |
| *Ranunculus alpestris* | 2283, 2379, 2587 |
| *Ranunculus montanus* | 1463, 1632, 1860, 2080, 2283, 2379 |
| *Ranunculus repens* | 1463 |
| *Rhinanthus alectorolophus* | 1181 |
| *Rhinanthus glacialis* | 2283 |
| *Rhododendron ferrugineum* | 1632, 1860, 2080 |
| *Rumex alpestris* | 1181, 1463, 1632, 1860, 2283 |
| *Rumex alpinus* | 1632, 1860 |
| *Sagina nodosa* | 1181 |
| *Saxifraga aizoides* | 2283, 2587 |
| *Saxifraga androsacea* | 2283 |
| *Saxifraga exarata* | 2379, 2587 |
| *Saxifraga oppositifolia* | 2587 |
| *Saxifraga paniculata* | 2283, 2379 |
| *Saxifraga rotundifolia* | 1632 |
| *Saxifraga stellaris* | 1860, 2283 |
| *Scabiosa columbaria* | 1181, 1463 |
| *Scorzoneroides autumnalis* | 1181, 1463, 1632, 1860 |
| *Sempervivum montanum* | 2080 |
| *Silene acaulis* | 2080, 2283, 2379, 2587 |
| *Silene dioica* | 1632, 2283 |
| *Silene nutans* | 1181, 1463 |
| *Silene vulgaris* | 1181, 1463, 1632, 2080, 2283 |
| *Soldanella pusilla* | 1860, 2080, 2283 |
| *Stellaria graminea* | 1181, 1463, 1632 |
| *Stellaria nemorum* | 1860 |
| *Taraxacum campylodes* | 1181, 1463, 1632, 1860 |
| *Thymus praecox* | 1181, 1463, 1632, 1860, 2080, 2283 |
| *Thymus pulegioides* | 1463, 1860 |
| *Tofieldia calyculata* | 2080, 2379 |
| *Trifolium badium* | 2080, 2283, 2379 |
| *Trifolium pallescens* | 2283, 2379 |
| *Trifolium pratense* | 1181, 1463, 1632, 1860, 2080, 2283, 2379 |
| *Trifolium repens* | 1181, 1463, 1632, 1860, 2080 |
| *Trollius europaeus* | 1632 |
| *Vaccinium myrtillus* | 1181, 1463, 1632, 1860, 2080 |
| *Vaccinium vitis idaea* | 1181, 1463, 1860, 2080 |
| *Veratrum album* | 2080 |
| *Veronica alpina* | 1632, 1860 |
| *Veronica chamaedrys* | 1181, 1463, 1860 |
| *Veronica officinalis* | 1181, 1463, 1632 |
| *Veronica serpyllifolia* | 1463, 1632, 1860 |
| *Viola biflora* | 1181, 1463, 1632, 1860, 2080 |
| *Viola canina* | 1181, 1463, 1632, 1860 |

**Table S3** List of floral hypervolume variants and the phenotypic traits included in the calculation of each. The first hypervolume includes all traits and the remaining variants included only a subset of those traits. Each hypervolume variant was defined to include traits to describe an aspect of floral traits. Note that some traits were included in more than one of the subset hypervolumes

| **Floral Traits** |  | **Hypervolume Variants** | | | | | |
| --- | --- | --- | --- | --- | --- | --- | --- |
|  |  | **All Floral Traits** | **Floral Display (Except Color)** | **Morphology** | **Nectary-Specific** | **Pollen** | **Color Only** |
| **Display size** |  | X | X |  |  |  |  |
| **Flower diameter** |  | X | X | X |  |  |  |
| **Floral depth** |  | X | X | X |  |  |  |
| **Floral width** |  | X | X |  |  |  |  |
| **Petal length** |  | X | X |  |  |  |  |
| **Stylus length** |  | X |  |  |  |  |  |
| **Filament length** |  | X |  |  |  | X |  |
| **Nectar depth** |  | X |  | X | X |  |  |
| **Nectar width** |  | X |  | X | X |  |  |
| **Stylus position** |  | X |  |  |  |  |  |
| **Filament position** |  | X |  |  |  | X |  |
| **Plant height** |  | X | X |  |  |  |  |
| **Flower inclination** |  | X | X |  |  |  |  |
| **Number of inflorescences** |  | X | X |  |  |  |  |
| **Number of flowers per inflorescence** |  | X | X |  |  |  |  |
| **Color (s)** |  | X |  |  |  |  | X |
| **Color (m)** |  | X |  |  |  |  | X |
| **Color (l)** |  | X |  |  |  |  | X |
| **Color (lum)** |  | X |  |  |  |  | X |

**Table S4** Summary statistics for correlations elevation and network structure indices. Degree column represents whether best model includes a quadratic or cubic elevation term. Boldface depicts statistically significant relationships (*p <* 0.05)

| **Network Index** |  | ***F*** | ***p*** | ***adjusted-r^2^*** | ***degree*** |
| --- | --- | --- | --- | --- | --- |
| H_2_' |  | **5.939** | **0.005** | **0.392** | **3** |
| Modularity Q |  | **8.464** | **0.001** | **0.493** | **3** |
| Weighted NODF (Nestedness) |  | **14.400** | **0.001** | **0.368** | **1** |

**Table S5** Summary statistics for relationships between network indices and unweighted floral trait hypervolumes. Hypervolume variants refer to unweighted hypervolumes. No relationships are statistically significant (*p <* 0.05)

| **Hypervolume Variant (Weighted)** |  | **H_2_'** | | |  | **Modularity Q** | | |  | **Weighted NODF** | | |
| --- | --- | --- | --- | --- | --- | --- | --- | --- | --- | --- | --- | --- |
|  |  | ***t*** | ***p*** | ***r^2^*** |  | ***t*** | ***p*** | ***r^2^*** |  | ***t*** | ***p*** | ***r^2^*** |
| All Floral Traits |  | 0.539 | 0.595 | 0.013 |  | 0.812 | 0.425 | 0.029 |  | **-2.338** | **0.029** | **0.199** |
| Display (No Color) |  | 0.287 | 0.777 | 0.004 |  | 0.300 | 0.767 | 0.004 |  | **-2.234** | **0.036** | **0.185** |
| Morphology |  | 0.625 | 0.538 | 0.017 |  | 1.009 | 0.324 | 0.044 |  | -0.981 | 0.337 | 0.042 |
| Nectary |  | 0.912 | 0.372 | 0.036 |  | 1.377 | 0.182 | 0.079 |  | -0.759 | 0.456 | 0.026 |
| Pollen |  | 0.642 | 0.527 | 0.018 |  | 1.471 | 0.156 | 0.089 |  | -1.694 | 0.104 | 0.115 |
| Color |  | -0.373 | 0.713 | 0.006 |  | 0.796 | 0.434 | 0.028 |  | -1.884 | 0.073 | 0.139 |

**Fig. S1** Contrasting qualitative and quantitative measures of functional diversity. We illustrate how qualitative and quantitative functional diversity measures differ by comparing two flowering plant communities characterized by the abundance of member species (i.e., area of circle) and their position in a niche space defined along two axes (i.e., nectar tube depth, flower size). **A)** In this example, member species of Community 1 (red circles) and Community 2 (blue circles) occupy niche spaces with similar ranges of values. However, these two communities differ in where in niche space the most abundant species are located, and this is reflected in their measures of functional diversity. **B)** Community 1 is less functionally diverse than Community 2 as measured by *unweighted* hypervolumes. This results from species in Community 1 being clustered over a smaller area in comparison with species in Community 2, which are more dispersed (note the species located in the bottom- and top-right in panel A). **C)** Contrastingly, Community 1 is functionally diverse than Community 2 as measured by *weighted* hypervolumes. This results from taking into account species abundance, which increases the influence of the common species’ traits in the calculation of the hypervolumes. Notice that while the most abundant species in Community 1 have highly dissimilar traits, the most abundant species in Community 2 have traits that are more similar to each other

**Fig. S2** Heatmap visualizing the correlations between elevation, indices of diversity and network structure. Correlations values are calculated using the *cor.mtest()* function in the corrplot R package (Wei and Simko 2017). Statistically significant correlations are depicted in color, negative correlations in red and positive correlations in blue. Refer to Appendix Table S3 for description of hypervolume variants

**Fig. S3** Hypothesized SEM’s for floral diversity (left) and pollinator diversity (right)

Literature Cited

Wei, T, Simko, V (2017) R package "corrplot": Visualization of a correlation matrix.R package version 0.84:.

stylefix
